# Spatial and functional targeting of intratumoral Tregs reverses CD8^+^ T cell exhaustion and promotes cancer immunotherapy

**DOI:** 10.1172/JCI180080

**Published:** 2024-05-23

**Authors:** Lei Zhou, Maria Velegraki, Yi Wang, J K Mandula, Yuzhou Chang, Weiwei Liu, No-Joon Song, Hyunwoo Kwon, Tong Xiao, Chelsea Bolyard, Feng Hong, Gang Xin, Qin Ma, Mark P. Rubinstein, Haitao Wen, Zihai Li

**Affiliations:** 1 Pelotonia Institute for Immuno-Oncology (PIIO), The Ohio State University Comprehensive Cancer Center – James Cancer Hospital and Solove Research Institute (OSUCCC), Columbus, Ohio, USA.; 2 Department of Anesthesiology, The Third Xiangya Hospital, Central South University, Changsha, Hunan, China.; 3 Molecular, Cellular and Developmental Biology Graduate Program, Ohio State University, Columbus, Ohio, USA.; 4 Department of Biomedical Informatics,; 5 Department of Internal Medicine, Ohio State University College of Medicine, Columbus, USA.; 6 Division of Medical Oncology, Department of Internal Medicine, Ohio State University Comprehensive Cancer Center, Columbus, USA.; 7 Department of Microbial Infection and Immunity, Ohio State University College of Medicine, Columbus, USA.

**Keywords:** Immunology, Oncology, Cancer immunotherapy, Chaperones, T cells

## Abstract

Intratumoral Tregs are key mediators of cancer immunotherapy resistance, including anti–programmed cell death (ligand) 1 [anti–PD-(L)1] immune checkpoint blockade (ICB). The mechanisms driving Treg infiltration into the tumor microenvironment (TME) and the consequence on CD8^+^ T cell exhaustion remain elusive. Here, we report that heat shock protein gp96 (also known as GRP94) was indispensable for Treg tumor infiltration, primarily through the roles of gp96 in chaperoning integrins. Among various gp96-dependent integrins, we found that only LFA-1 (αL integrin), and not αV, CD103 (αE), or β7 integrin, was required for Treg tumor homing. Loss of Treg infiltration into the TME by genetic deletion of gp96/LFA-1 potently induced rejection of tumors in multiple ICB-resistant murine cancer models in a CD8^+^ T cell–dependent manner, without loss of self-tolerance. Moreover, gp96 deletion impeded Treg activation primarily by suppressing IL-2/STAT5 signaling, which also contributed to tumor regression. By competing for intratumoral IL-2, Tregs prevented the activation of CD8^+^ tumor-infiltrating lymphocytes, drove thymocyte selection-associated high mobility group box protein (TOX) induction, and induced bona fide CD8^+^ T cell exhaustion. By contrast, Treg ablation led to striking CD8^+^ T cell activation without TOX induction, demonstrating clear uncoupling of the 2 processes. Our study reveals that the gp96/LFA-1 axis plays a fundamental role in Treg biology and suggests that Treg-specific gp96/LFA-1 targeting represents a valuable strategy for cancer immunotherapy without inflicting autoinflammatory conditions.

## Abstract 2

Deleting gp96/LFA-1 blocks Treg accumulation in the tumor microenvironment and releases CD8^+^ T cells from exhaustion, resulting in potent antitumor immunity without induction of autoimmunity.

## Graphical abstract

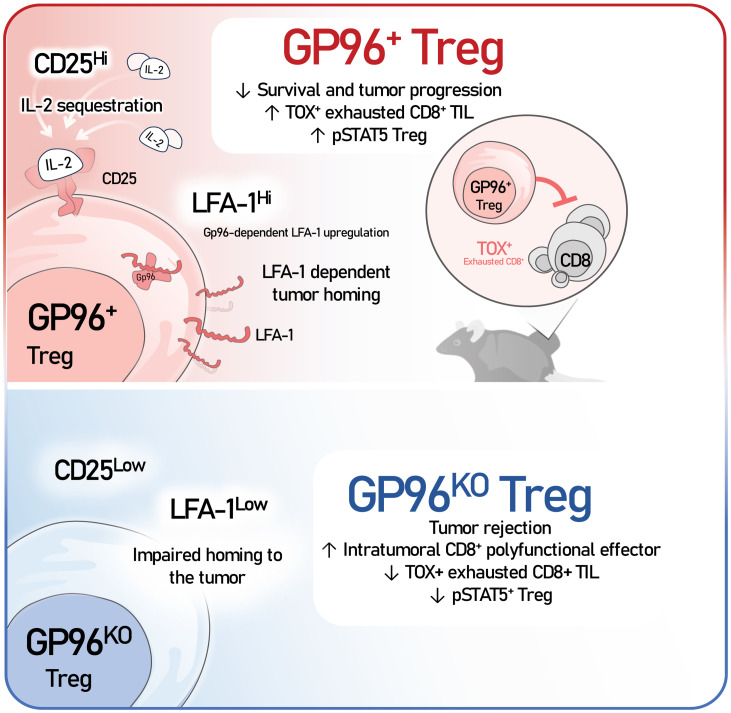

## Introduction

Regulatory T cells (Tregs) are characterized by high expression of IL-2 receptor α chain (CD25) and the transcription factor Foxp3, and their function in suppressing the effector immune response against self-antigens (1–3) and inhibiting antitumor immunity (4). Tregs represent a major barrier in cancer immunotherapy, given their ability to accumulate in the tumor microenvironment (TME) and suppress antitumor immune effector cells (5, 6). A reduction of intratumoural Tregs strongly correlates with clinical benefit in diverse cancer types, and Treg-directed therapy has been postulated as a promising anticancer therapy (7–10).

gp96/GRP94 is an endoplasmic reticulum (ER) master chaperone for various proteins including TLRs, integrins, and glycoprotein A repetitions predominant (GARP) (11). Our previous work demonstrated that gp96 is a critical mediator of Treg lineage stability, as its deletion resulted in downregulated Foxp3 expression and impaired Treg function (11, 12). However, it is unclear whether targeting Tregs via gp96 can reprogram the adaptive immunity against cancer to enhance immunotherapy without systemic toxicities.

Using mice with KO of tamoxifen-inducible, Treg-specific *Hsp90b1* (encoding gp96), we discovered that gp96 was required for Treg infiltration into the tumor. Treg-specific gp96 deletion abolishes Treg infiltration into the tumor and eradicates cancer without evidence of autoimmunity. Mechanistically, we demonstrated that gp96 and its client αL integrin (LFA-1) were indispensable for Treg migration into the TME. gp96 deletion also inhibited Treg activation, as evidenced by reduced levels of CD25, Foxp3, and other activation markers, attributed primarily to the downregulation of the IL-2/phosphorylated STAT5 (IL-2/p-STAT5) signaling pathway. Importantly, by genetically and pharmacologically targeting the gp96/LFA-1 axis, we found that loss of infiltrating Tregs in the TME potentiated the antitumor CD8^+^ T cell effector response and prevented Treg functional exhaustion in an IL-2-dependent manner. Collectively, these findings suggest that targeting gp96/LFA-1 axis–mediated Treg infiltration into the TME is a powerful strategy to augment anti–PD-1 cancer immunotherapy by overcoming CD8^+^ T cell exhaustion without inducing autoimmune diseases.

## Results

### Treg-specific gp96 deletion results in tumor regression and prolonged survival without disturbing immune homeostasis.

**Figure 1:**
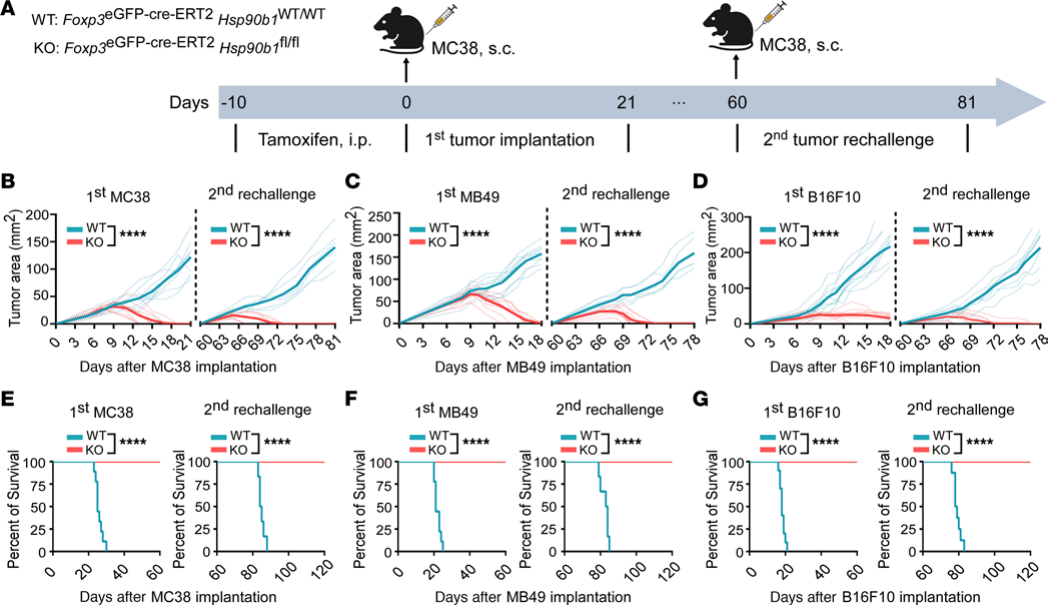
Treg-specific gp96 deletion results in tumor regression and prolonged survival in mice. (**A**) Experimental schema for primary implantation and rechallenge of MC38 tumor cells in *Foxp3^eGFP-Cre-ERT2^* *Hsp90b1^WT/WT^* (WT) and *Foxp3^eGFP-Cre-ERT2^* *Hsp90b1^fl/fl^* (KO) mice. For primary implantation, WT or KO mice (8–10 weeks old; *n* = 9/group) received tamoxifen for 10 days (75 mg/kg, i.p.; days –10 to 0), followed by a single s.c. injection of MC38 tumor cells (2 × 10^6^ cells/mouse; day 0) into their right flank. Tumor volumes were measured daily or every 2 days (length × width in mm) using a digital caliper, starting from day 5 after tumor cell implantation. For rechallenge, all tumor-regressed KO mice and age-matched tumor-naive WT mice were rechallenged s.c. on the opposite flank with 2 × 10^6^ MC38 tumor cells 60 days after primary tumor cell implantation. Tumor growth was monitored as described. (**B**–**D**) Growth curves depict primary implantation and rechallenge with 2 × 10^6^ MC38 (**B**), 1 × 10^6^ MB49 (**C**), and 2.5 × 10^5^ B16-F10 (**D**) tumor cells in WT and KO mice. MB49 and B16F10 tumors were implanted following the same scheme as that used for MC38 tumor cells. *n* = 6–10/group. (**E–G**) Survival curves following primary inoculation and rechallenge with MC38 (**E**), MB49 (**F**), and B16-F10 (**G**) tumor cells in WT and KO mice. Mice were euthanized when tumors reached more than 16 mm in diameter. *n* = 6–10/group. Results are representative of more than 3 independent experiments. Tumor growth curves were analyzed by repeated-measures, 2-way ANOVA (**B**–**D**); survival incidence analysis was performed by log-rank (Mantel-Cox) test (**E**–**G**); ****P < 0.0001 (KO vs. WT).

Tregs play a critical role in suppressing antitumor immunity, and targeting Tregs to enhance cancer immunotherapy holds great promise (13). We previously discovered that gp96 is critical for maintaining Treg homeostasis, as genetic deletion of *Hsp90b1* in Tregs in nonobese diabetic (NOD) mice leads to rapid, fatal inflammatory disease (11). We found that gp96-deficient Tregs downregulated *Foxp3* expression and ultimately converted to IFN-γ–producing “ex-Tregs” (11). To investigate the effect of Treg-specific gp96 deletion on tumor control in nonautoimmune-prone mice, we generated tamoxifen-inducible, Treg-specific gp96-KO mice (*Foxp3^eGFP-Cre-ERT2^* *Hsp90b1^fl/fl^*) on a C57BL/6 background and challenged them with several syngeneic tumor models including MC38/colon (Figure 1, A and B, and Supplemental Table 1; supplemental material available online with this article; https://doi.org/10.1172/JCI180080DS1); MB49/bladder (Figure 1C and Supplemental Table 1); and immune checkpoint blockade–resistant (ICB-resistant) B16-F10/melanoma (Figure 1D and Supplemental Table 1). Both WT (*Foxp3^eGFP-Cre-ERT2^* *Hsp90b1^WT/WT^*) and gp96-KO mice were treated with 75 mg/kg tamoxifen for 10 days, which led to effective gp96 deletion in Tregs from KO mice for at least 20 days (Supplemental Figure 1). Following tamoxifen treatment (days –10 to 0), mice were implanted s.c. with tumor cells (day 0) and followed for tumor growth and overall survival. Tumors grew progressively in the WT mice but were rejected completely in the KO mice. The rejection of B16-F10 tumors in the KO mice was especially noteworthy because this model is notoriously poorly immunogenic. To evaluate the generation of immunologic memory, the KO mice were rechallenged with tumor cells on day 60 in the absence of tamoxifen treatment; these mice remained completely protected (Figure 1, B–D, and Supplemental Table 1). In both the primary tumor cell implantations and tumor cell rechallenge, all KO mice had prolonged survival (100%), whereas WT mice succumbed to the tumors (Figure 1, E–G).

**Figure 2:**
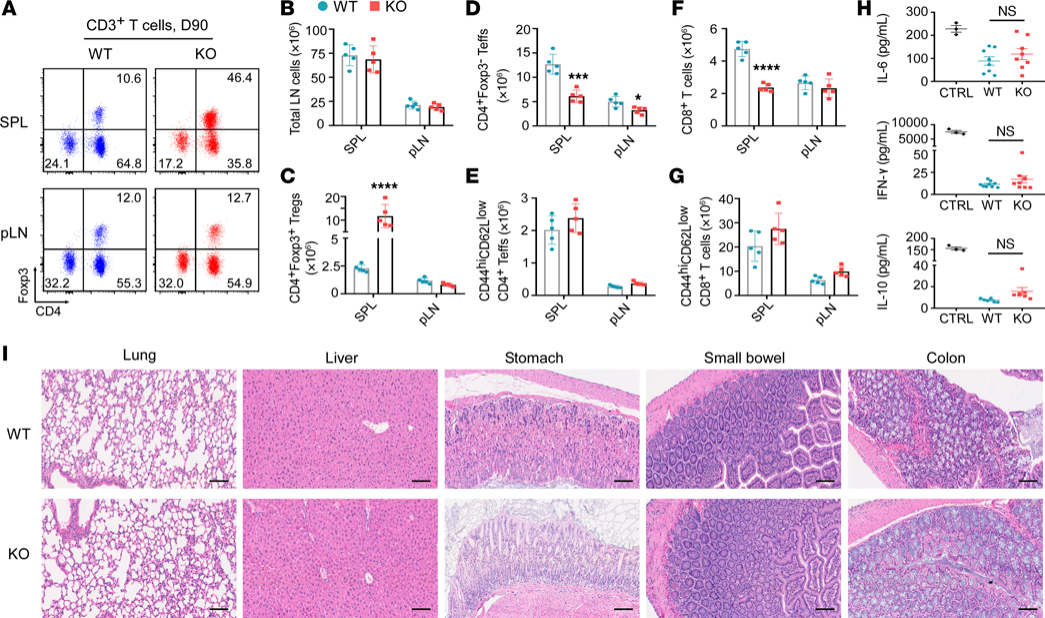
Treg-specific gp96 deletion preserves immune homeostasis in mice. Both WT and KO mice received a 10-day tamoxifen treatment (days –10 to 0). Ninety days later (D90), the mice were euthanized and specified tissues were collected for analysis. (**A**) Representative flow plots illustrate the distribution of CD3^+^ T cell subsets in murine SPLs and pLNs from both groups (*n* = 5/group), with values indicating the percentages of the specified subsets in total CD3^+^ T cells. (**B**–**G**) Absolute numbers of total lymphocytes (total LN cells) (**B**), CD4^+^Foxp3^+^ Tregs (**C**), CD4^+^Foxp3^–^ Teffs (**D**), CD44^hi^CD62L^lo^CD4^+^ Teffs (**E**), CD8^+^ T cells (**F**), and CD44^hi^CD62L^lo^CD8^+^ T cells (**G**) in SPLs and pLNs from mice of both groups. (**H**) Serum levels of IL-6, IFN-γ, and IL-10 were measured on day 90 following tamoxifen administration (days –10 to 0) in WT and KO mice using ELISA. *n* = 6–9/group. CTRL, positive control. (**I**) Representative H&E-stained images of the indicated organs from WT and KO mice (day 90; *n* = 5–8/group). Scale bars: 100 μm. Results are representative of more than 3 independent experiments. Data are shown as the mean ± SEM. *P < 0.05, ***P < 0.001, and ****P < 0.0001 (KO vs. WT). For statistical analyses, a 2-tailed, unpaired Student’s *t* test was performed (**B**–**H**).

Targeting Tregs for cancer immunotherapy confers the risk of eliciting systemic autoimmune diseases (14). Both humans and mice can develop various forms of autoimmune diseases upon genetic or pharmacologic inhibition of Tregs (4, 5, 15–17). Unexpectedly, we found that deletion of gp96 from Tregs in adult mice did not result in overt inflammation or autoimmune diseases for at least 3 months (Figure 2 and Supplemental Figure 2). We characterized the immune phenotype of effector T cells (Teffs) from the spleens (SPLs) and peripheral lymph nodes (pLNs) of these long-surviving mice (Figure 2, A–G). The total numbers of lymphocytes in both the SPL and pLNs were comparable between WT and KO mice (Figure 2B), however, their cellularity was distinct in the SPL. Upon gp96 deletion, splenic Tregs expanded, but the non–Treg T cell population dropped in both frequency and absolute number (Figure 2, C, D, and F). Hence, although the relative frequencies of activated CD44^hi^CD62L^lo^CD4^+^Teffs and CD44^hi^CD62L^lo^CD8^+^ T cells increased in the KO mice (Supplemental Figure 2, A and B), the absolute numbers of both subsets remained the same (Figure 2, E and G). In addition, KO mice did not have abnormal levels of systemic cytokines such as IL-10, IL-6, and IFN-γ (Figure 2H). To rule out subclinical organ inflammation in KO mice, we sacrificed KO mice and performed necropsy; this demonstrated no obvious infiltration by neutrophils or lymphocytes in any of the organs examined (Figure 2I). The comparable body weights between WT and KO mice also indicated that Treg-specific gp96 deletion did not lead to the development of subclinical autoimmune diseases (Supplemental Figure 2C). Collectively, these data suggest that Treg-specific gp96 deletion in adult mice results in vigorous eradication of various tumors and extends survival without disturbing immune homeostasis, indicating that gp96 is a promising candidate for Treg-targeted therapy against cancers.

### The Gp96/LFA-1 axis is required for Treg infiltration into the TME.

**Figure 3:**
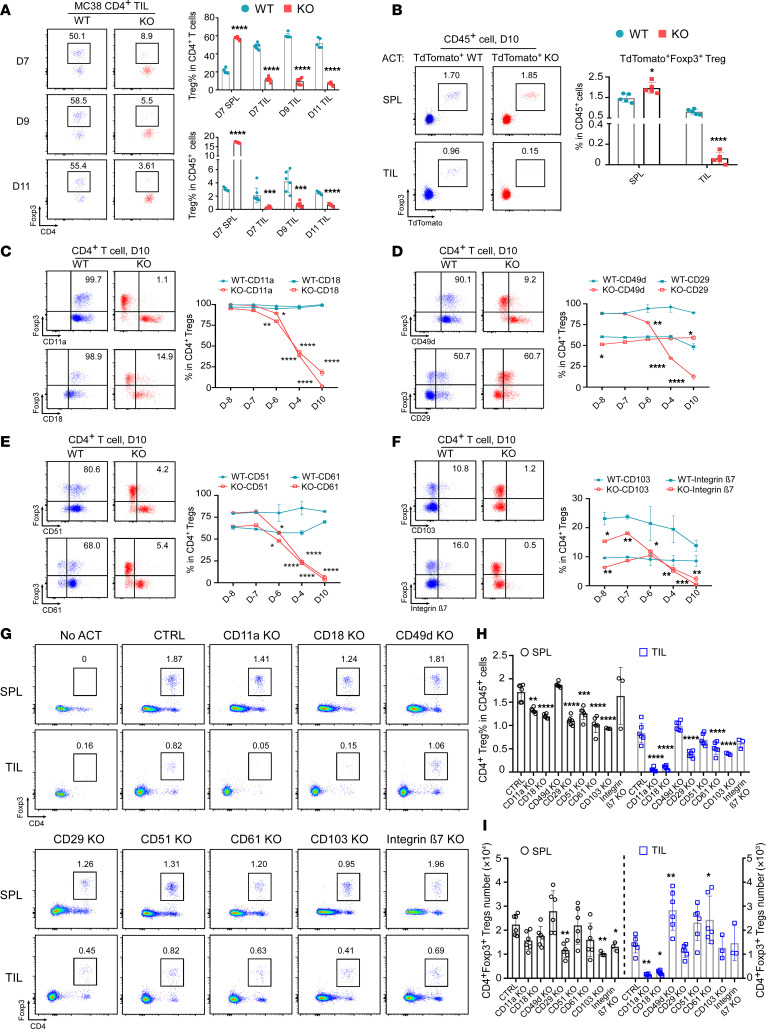
Gp96 regulates CD11a/CD18 (LFA-1) integrin expression in Tregs and facilitates their infiltration into the TME. (**A**) WT and KO mice (*n* = 5–8/group) were pretreated with tamoxifen (days –10 to 0) and s.c. implanted with MC38 tumors on day 0. TILs were harvested on days 7, 9, and 11. Representative flow cytometric plots and summary graphs show the percentages of CD4^+^Foxp3^+^ Tregs in specified tissues at these time points. (**B**) *Rag2^–/–^* recipient mice (*n* = 5/group) were implanted s.c. with 2 × 10^6^ MC38 cells on day 0. TdTomato-expressing Tregs from SPLs of *R26^STOP-tdTomato^* *Foxp3^eGFP-Cre-ERT2^* *Hsp90b1^WT/WT^* (TdTomato-WT) or *R26^STOP-tdTomato^* *Foxp3^eGFP-Cre-ERT2^* *Hsp90b1^fl/fl^* (TdTomato-KO) donor mice were collected, preactivated, and transferred (2 × 10^6^ cells/mouse; *n* = 5/group) into recipient mice on day 3. On day 10, SPLs and tumors were harvested for flow cytometry. Representative flow cytometric plots and summary graphs indicate the percentages of infiltrating TdTomato^+^Foxp3^+^ Tregs among CD45^+^ cells. (**C**–**F**) WT and KO mice (*n* = 3/group) received tamoxifen (days -10 to 0), and splenic Tregs’ integrin expression was assessed using flow cytometry at designated time points (D–8, day –8; D–7, day –7; D–6, day –6; D–4, day –4; D10, day 10). Representative flow cytometric plots (day 10) and summary graphs (time course) show frequencies of indicated surface integrins on splenic Foxp3^+^ Tregs in WT and KO mice. (**G**–**I**) Naive CD4^+^ T cells from SPLs of C57BL/6 mice were differentiated into iTregs under Treg-skewed conditions for 2 days (days –2 to 0) followed by CRISPR/Cas9 KO of indicated integrins on day 0; cells were cultured for 3 more days (days 0–3). MC38 tumor–bearing *Rag2^–/–^* mice (*n* = 3–6/group) received specific integrin-KO or nontargeting control iTregs on day 3 post-tumor implantation; SPLs and tumors were collected on day 10 for flow cytometry. Representative flow cytometric plots (**G**) and summary graph (**H**) show relative number of Foxp3^+^ Tregs (in CD45^+^ cells total). (**I**) Absolute numbers of Tregs in SPLs and TILs. Results represent 3 independent experiments. Data indicate the mean ± SEM. *P < 0.05, **P < 0.01, ***P < 0.001, and ****P < 0.0001 (KO vs. WT), by 2-tailed Student’s *t* test used for comparisons of different experimental groups (**A**–**F**) and 1-way ANOVA with Dunnett’s multiple-comparison test for multiple-comparison analyses (**H** and **I**).

Next, we collected tumor-infiltrating lymphocytes (TILs) from MC38 and MB49 cells grown in WT and KO mice to examine the underlying mechanism. Strikingly, we found very few Tregs in the MC38 or MB49 TME, even at very early stages following gp96 deletion (Figure 3A and Supplemental Figure 3), suggesting that gp96 deletion limited either Treg survival or TME recruitment. We considered whether gp96-null Tregs retained the ability to migrate into the tumor but converted into so-called Foxp3^–^ “ex-Tregs” in the TME. To evaluate this possibility, we crossed the Treg-specific gp96-KO mice with Ai14 reporter mice that express tdTomato following Cre recombination (R26STOP-tdTomato), generating mice that produce tdTomato-labeled gp96-KO Tregs upon tamoxifen treatment (*R26^STOP-tdTomato^* *Foxp3^eGFP-Cre-ERT2^* *Hsp90b1^fl/fl^*, referred to herein as TdTomato-KO mice). Similar to gp96-KO mice, these TdTomato-KO mice, after a 10-day tamoxifen treatment, showed complete rejection of MC38 (Supplemental Figure 4, A and B, and Supplemental Table 2). However, we detected very few Foxp3^–^TdTomato^+^ or Foxp3^+^TdTomato^+^ Tregs in the MC38 TME (Supplemental Figure 4, C and D), suggesting that gp96-null Tregs did not become ex-Tregs, but lost their ability to migrate into the tumor. To confirm this possibility, we performed an adoptive T cell transfer and fate-mapping experiment. Tamoxifen was administrated for 10 days to TdTomato-WT (*R26^STOP-tdTomato^* *Foxp3^eGFP-Cre-ERT2^* *Hsp90b1^WT/WT^*) and TdTomato-KO donor mice. Tregs were isolated from SPLs, preactivated, and adoptively transferred into MC38-bearing *Rag2^–/–^* recipient mice. On day 10, we found that TdTomato^+^ Treg frequencies in the SPL were comparable between the WT and KO groups, but tdTomato^+^ gp96-KO Tregs were significantly reduced in the TILs (Figure 3B). These findings suggest that gp96 is indispensable for Treg migration into the TME.

gp96 is known for the folding and cell-surface expression of selected integrins including integrins αl, β2, α4, and αV (18). Since integrins facilitate immune cell adhesion and transmigration into tissues (19), as well as mediate Treg function in controlling colitis (20), we hypothesized that Treg TME migration is also controlled by integrins. Using flow cytometry, we profiled in WT and KO Tregs the expression of several paired integrins, including CD11a(αL)/CD18(β2), CD49d(α4)/CD29(β1), CD51(αv)/CD61(β3), and CD103(αe)/integrin β7. As expected, since CD29 is not a client of gp96, we saw no reduction of its expression on the cell surface of KO Tregs (Figure 3D). Other integrins, including CD11a, CD18, CD49d, CD51, CD61, CD103, and integrin β7, began to decrease by day –6 and were nearly absent on day –4 during tamoxifen treatment (Figure 3, C–F, and Supplemental Figure 5). Chemokine receptors such as CCR4 are involved in the trafficking and recruitment of Tregs (21, 22). However, we found that gp96-KO Tregs expressed similar levels of CCR7, CCR6, CCR2, CX3CR1, and CXCR5 and even higher levels of CCR4, CCR9, and CXCR3 (Supplemental Figure 6), suggesting that gp96 controls Treg migration primarily through integrins rather than chemokine receptors.

**Figure 4:**
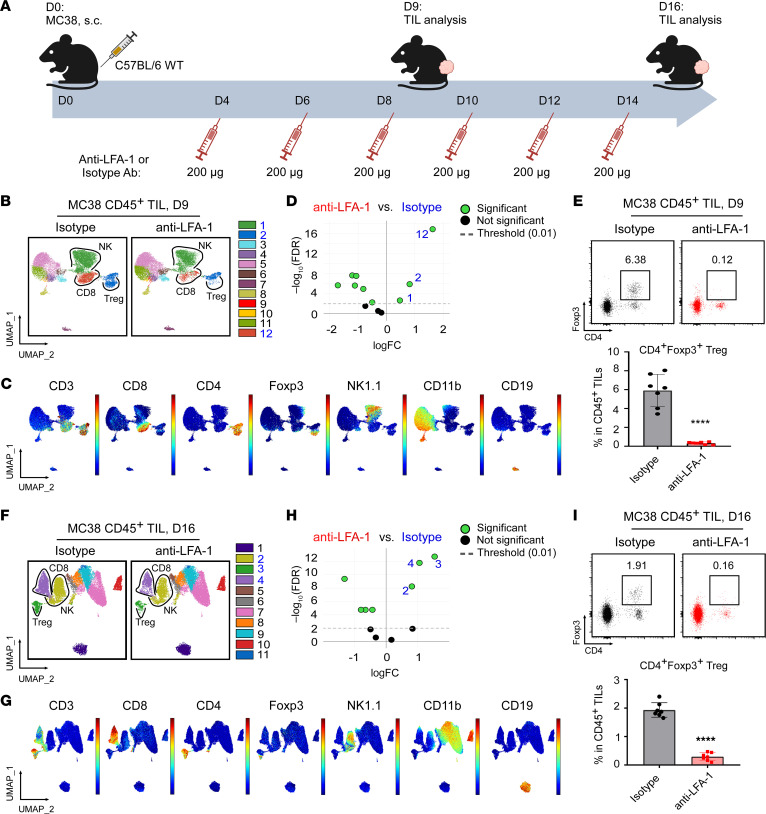
LFA-1 blockade prevents Treg infiltration into the TME. (**A**) Experimental scheme illustrates the process of LFA-1 blockade using anti–LFA-1 or IgG2a isotype-matched control Abs in C57BL/6 mice implanted with MC38 tumor cells. Anti–LFA-1 or isotype Abs were administered every 2 days starting from day 4 after MC38 tumor cell implantation on day 0; TIL analysis was conducted on day 9 (*n* = 7/group) and day 16 (*n* = 7/group). (**B**–**D**) Spectral flow cytometric analysis of CD45^+^ TILs from day-9 MC38 tumors treated with anti–LFA-1 or isotype Abs. LFA-1 blockade significantly reduced the frequency of cluster 1 (NK cells), cluster 2 (including CD3^+^CD4^+^Foxp3^–^ non-Tregs and CD3^+^CD4^+^Foxp3^+^ Tregs), and cluster 12 (CD8^+^ T cells) (highlighted in blue). As a subset of CD4^+^ T cells, Tregs expressed high levels of Foxp3 and were located at the bottom of cluster 2. (**C**) UMAP visualization shows the distribution of the indicated markers. (**D**) edgeR analysis indicating CD45^+^ TILs clusters with significant changes to frequency following treatment with anti–LFA-1 (left) versus isotype Abs (right). (**E**) Representative flow cytometric plots and graph depict the percentages of Foxp3^+^ Tregs in CD45^+^ TILs from day-9 MC38 tumors. (**F**–**H**) Spectral flow cytometric analysis of CD45^+^ TILs from day-16 MC38 tumors treated with anti–LFA-1 or isotype Abs. Similar to results in **B**, cluster 2 (NK cells), cluster 3 (including CD3^+^CD4^+^Foxp3^–^ non-Tregs and CD3^+^CD4^+^Foxp3^+^ Tregs), and cluster 4 (CD8^+^ T cells) exhibited reduced abundance following LFA-1 blockade (highlighted in blue). (**F**) FoxP3^+^ Tregs were localized at the bottom of cluster 3. (**G**) UMAP visualization shows the distribution of the indicated markers. (**H**) edgeR analysis indicating CD45^+^ TILs clusters with significant changes to frequency following anti–LFA-1 versus isotype Ab treatment. (**I**) Representative flow cytometric plots and graph depict the percentages of Foxp3^+^ Tregs in CD45^+^ TILs from day-16 MC38 tumors. Results are representative of 3 independent experiments. Data are shown as the mean ± SEM. ****P < 0.0001 (anti–LFA-1 vs. isotype), by 2-tailed Student’s *t* test for comparisons of different experimental groups (**E** and **I**). FC, fold change.

To determine which integrins play a role in modulating Treg TME infiltration, we next genetically deleted various integrins from in vitro–differentiated induced Tregs (iTregs) via CRISPR/Cas9, followed by adoptive transfer into *Rag2^–/–^* mice bearing MC38 tumors. We confirmed the efficiency of the deletion of integrins before transfer (>80%; Supplemental Figure 7). On day 10, we found that infiltration of Tregs into the TME was almost completely abolished after deletion of CD18/CD11a (also called LFA-1) (Figure 3, G–I). By comparison, Treg TME infiltration was either not significantly affected or substantially reduced by deleting other integrins, including CD51 (αV), CD103 (αE), CD29 (β1), CD61 (β3), or integrin β7. To confirm that LFA-1 is required for Treg TME infiltration, we performed an in vivo experiment using an anti–LFA-1–blocking Ab (Figure 4A). Following systemic LFA-1 blockade, we noted a significant decrease in intratumoral Tregs on day-9 and day-16 MC38 tumors (Figure 4, B–I). Furthermore, CD8^+^ T cells and NK cells were also decreased in frequency at both time points, whereas anti-LFA-1 treatment induced minimal change in the frequencies of CD4^+^ Teffs, macrophages, neutrophils, and B cells. Reduced CD8^+^ T cells and NK cells in the TME could have been due to inactivation and inhibited proliferation upon LFA-1 blockade (23–25). Taken together, we conclude that gp96 promoted Treg trafficking into the TME largely through LFA-1. Although LFA-1 expression was previously shown to mediate gut tolerance by Tregs (26), to our knowledge, our study is the first to demonstrate important roles of LFA-1 in mediating Treg infiltration into the tumors.

To further understand the role of LFA-1 in mediating Treg trafficking into tumors in human cancers, we compared the transcriptional profiles of various α integrins in tumor-infiltrating and peripheral (PBMC) Tregs isolated via FACS from patients with breast cancer (Gene Expression Omnibus [GEO] GSE89225) (27). We found that LFA-1 (encoded by *ITGAL*) exhibited the highest expression among all α integrins in tumor-infiltrating Tregs (Supplemental Figure 8A). Moreover, breast cancer–infiltrating Tregs showed markedly higher expression of *ITGAL* compared with peripheral Tregs from the same patient cohort (Supplemental Figure 8A). We also performed immune deconvolution analyses of bulk RNA-Seq data accessed from The Cancer Genome Atlas (TCGA) to assess the differential retention of diverse immune cell populations between tumors with low and high levels (bottom quartile versus upper quartile, respectively) of LFA-1 in multiple cancer types (28). We found that elevated LFA-1 expression levels significantly correlated with increased Treg infiltration in these cancers (Supplemental Figure 8, B and C). However, minimal differences were observed in other immune cell subsets, such as NK cells, CD4^+^ Teffs, and myeloid subsets, based on LFA-1 expression (Supplemental Figure 8B). Furthermore, a negative association was found between high levels of LFA-1 and poorer survival in patients with solid cancers, including colorectal adenocarcinoma, uveal melanoma, lower grade glioma, or renal cell carcinoma (Supplemental Figure 8, D–G). Overall, our findings strongly support the notion that Tregs primarily rely on LFA-1 for their TME infiltration, thereby promoting immune evasion and contributing to poor clinical outcomes.

### gp96 deletion prevents full activation of effector Tregs by suppressing the IL-2/p-STAT5 signaling pathway.

**Figure 5:**
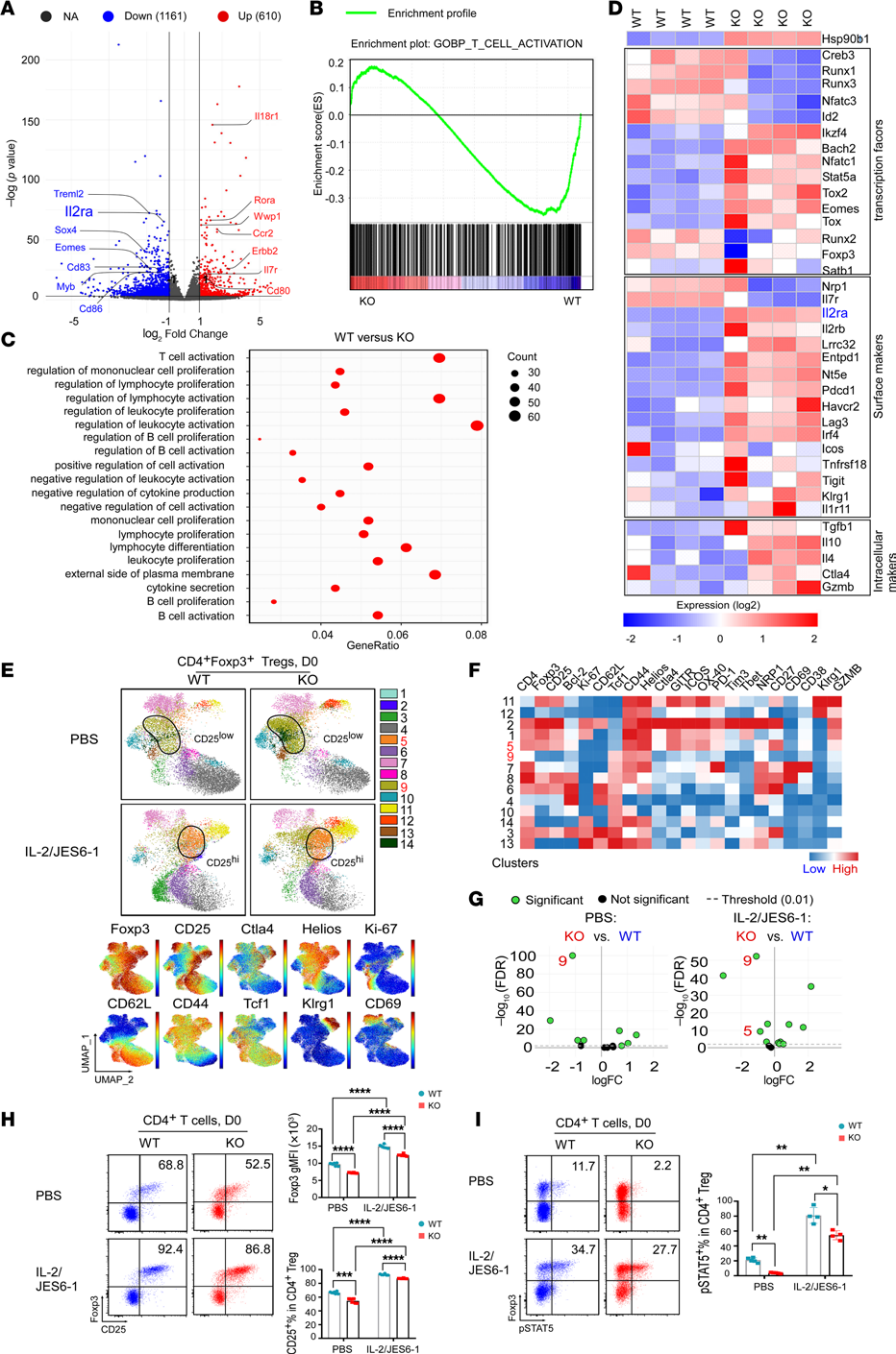
Gp96 deletion in Tregs reduces their CD25 expression but preserves their responsiveness to IL-2/p-STAT5 signaling activation. (**A**) Volcano plot depicting DEGs in splenic Tregs from KO versus WT mice after 10-day tamoxifen treatment (days –10 to 0; *n* = 4/group). The x axis indicates log2 fold change, and the y axis represents –log_10_ (corrected *P* value). Gray dots (NA) indicate no significant difference; blue dots (down) indicate downregulated genes in KO Tregs (adjusted *P* < 0.05; Ilr2a highlighted); red dots (up) indicate upregulated genes (adjusted *P* < 0.05). (**B**) GSEA of T cell activation genes between gp96-KO and WT Tregs shows FDR *q* = 0.03 and NES = –1.45. (**C**) GO enrichment scatter plots show the top 20 enriched pathways for DEGs in WT vs. gp96-KO Tregs, with dot size representing gene counts and GeneRatio indicating DEG ratios. (**D**) Heatmap of relative expression of selected genes encoding transcription factors, surface markers, and intracellular molecules in Tregs from WT and KO mice. Red indicates a high expression level; blue indicates a low expression level. Expression of *Ilr2a* (highlighted in blue) was significantly downregulated in gp96-KO Tregs. (**E**) UMAP visualization of splenic Tregs from WT and KO mice (top; *n* = 6–7/group). Mice received either PBS or IL-2/JES6-1 complex at specific time points, concurrently with tamoxifen treatment (days –10 to 0). Cluster 5 (CD25^hi^ Tregs) and cluster 9 (CD25^lo^ Tregs) were enriched in gp96-KO Tregs (highlighted in red). (**F**) Heatmap shows marker expression levels by cluster, with clusters 5 and 9 highlighted. (**G**) edgeR analysis indicates clusters significantly altered in splenic Tregs from WT (right) versus KO (left) mice receiving PBS or IL/JES6-1. Clusters 5 and 9 are highlighted. (**H**) Representative flow cytometric plots and summary graphs depict Foxp3 and CD25 expression in splenic Tregs from WT and KO mice on day 0, as described in **E**. Numbers indicate the frequencies of CD25^+^ subset in Foxp3^+^ Tregs. (**I**) Representative flow cytometric plots and graph depicting the ex vivo levels of p-STAT5 in splenic Tregs from WT and KO mice (*n* = 4–5/group) on day 0, as described in **E**. Numbers indicate frequencies of the p-STAT5^+^ subset in Foxp3^+^ Tregs. Results represent 3 independent experiments. Data are shown as the mean ± SEM. *P < 0.05, **P < 0.01, ***P < 0.001, and ****P < 0.0001, by 1-way ANOVA with Dunnett’s T3 multiple-comparison test (**H** and **I**).

We reported previously that gp96 is a critical mediator of Treg lineage stability (11, 12). Here, we performed RNA-Seq on splenic Tregs from KO and WT mice to assess gene expression profiles. Among all transcripts identified (*n* = 25,501), 610 were upregulated and 1,161 were downregulated in gp96-null Tregs (Figure 5A and Supplemental Table 3). Using gene set enrichment analysis (GSEA) and Gene Ontology (GO) study of differentially expressed targets, we found that the T cell activation pathway was most significantly downregulated in gp96-KO versus WT Tregs (Figure 5, B and C). For example, transcripts such as *Treml2,* *Il2ra,* *Sox4*, *Eomes*, *Cd83*, *Myb*, and *Cd86* were decreased in KO Tregs (Figure 5, A and B), suggesting that gp96 is required for Treg activation (29, 30), consistent with our previous work demonstrating that gp96 promotes optimal Ca^2+^ mobilization upon T cell receptor (TCR) engagement (31). We further analyzed the expression of Treg-related transcription factors, cell-surface markers, and intracellular markers (Figure 5D). Consistently, key Treg signature genes such as *Il2ra*, *Bach2*, *Stat5*, *Lrrc32*, and *Tgfb1* were downregulated in gp96-KO Tregs (Figure 5D). Since IL-2 controls Treg suppression function, differentiation, and lineage stability (1–3), we hypothesized that suboptimal IL-2 signaling may contribute to defects of gp96-KO Tregs.

IL-2, in complex with anti–IL-2 Ab JES6-1, activates IL-2/p-STAT5 signaling and increases CD25 and Foxp3 expression in Tregs (32); thus, we administered these complexes in tandem with a tamoxifen regimen to WT and gp96-KO mice. We observed that both CD25 and Foxp3 were decreased in gp96-KO Tregs (Figure 5, E–H). In particular, the CD25^lo^Foxp3^int^ subset (cluster 9) was markedly enriched in splenic Tregs of the KO mice, primarily due to the elevated proliferation of these cells (Supplemental Figure 9A). The expression of other activation markers such as CTLA4, CD69, and CD39 was also downregulated in gp96-KO Tregs (Figure 5, E–H, and Supplemental Figure 9, B–D). Ultimately, those KO Tregs had a reduced ability to suppress the proliferation of CD4^+^ Teffs and CD8^+^ T cells in vitro (Supplemental Figure 9, E and F). Of note, even though there was a concomitant reduction of ex vivo p-STAT5 levels in splenic KO Tregs, they remained responsive to high-dose IL-2 (Figure 5I). When treated with IL-2–JES6-1 complexes in vivo or rhIL-2 in vitro, gp96-KO Tregs exhibited enhanced STAT5 phosphorylation and regained high levels of Foxp3 and CD25 (cluster 5; Figure 5, E–I, and Supplemental Figure 10). The suboptimal IL-2 response by the gp96-KO Tregs was likely due to an integrin defect due to a known positive crosstalk between integrin signaling and IL-2 responsiveness (33, 34).

Given the reduced immunosuppressive function of gp96-KO Tregs, we sought to determine whether deletion of gp96 in Tregs has therapeutic benefit against preexisting tumors. To this end, we preestablished MC38 tumors to allow Treg infiltration into the TME, followed by conditional deletion of gp96 in Tregs (Supplemental Figure 11A). This maneuver indeed resulted in better tumor control without significantly affecting the number of Tregs in the TME (Supplemental Figure 11, B–F, and Supplemental Table 4), suggesting that targeting gp96 in Tregs may have therapeutic and translational potential.

### Intratumoral Tregs enhance CD8^+^ TIL thymocyte selection-associated high mobility group box protein (TOX) expression and promote functional exhaustion.

**Figure 6:**
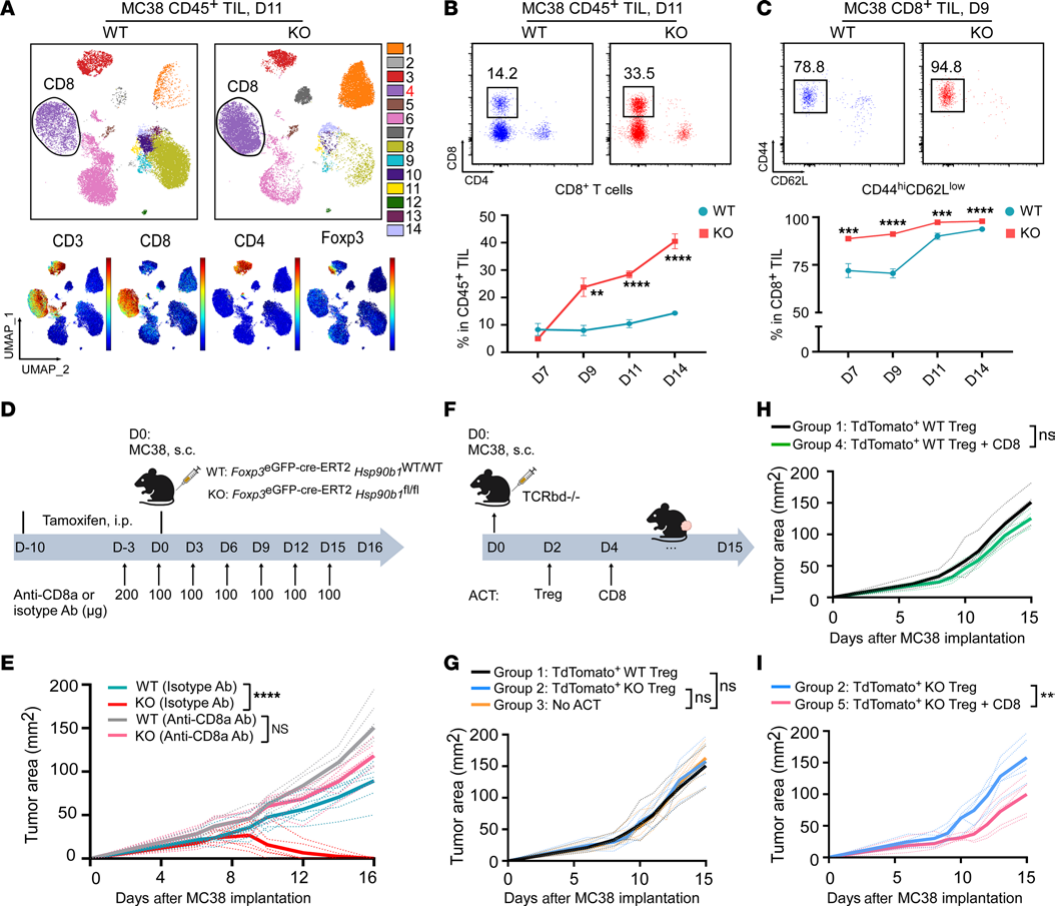
Upon gp96 deletion, the absence of infiltrating Tregs promotes CD8^+^ TIL accumulation and activation, leading to repression of MC38 tumors. (**A**) Spectral flow cytometric analysis of CD45^+^ TILs collected from day-11 MC38 tumors (implanted at 2 × 10^6^ cells on day 0) from WT and KO mice pretreated with tamoxifen (days –10 to 0; *n* = 6/group). Cluster 4 (CD8^+^ T cells; highlighted in red) expressing both CD3 and CD8 was significantly enriched in KO mice. Expression distribution of the indicated markers is shown in the bottom plots. (**B**) Representative flow cytometric plots (day 11) and summary graph (days 7, 9, 11, and 14) show the percentages of CD8^+^ T cells in CD45^+^ TILs from MC38 tumors from WT and KO mice (*n* = 5–8/group). (**C**) Representative flow cytometric plots (day 9) and summary graphs (days 7, 9, 11, and 14) show the frequencies of the CD44^hi^CD62L^lo^ population within CD8^+^ TILs from MC38 tumors grown in WT and KO mice (*n* = 5–8/group). (**D**) Experimental schema depicts the administration of CD8-depleting Ab (anti-CD8a Ab) or matched isotype Ab treatment in MC38 tumor–bearing WT and KO mice (2 × 10^6^ MC38 cells/mouse, s.c.; *n* = 6–7/group) that received tamoxifen before treatment (days –10 to 0). (**E**) Tumor growth curves of MC38 cells grown in WT and KO mice receiving anti-CD8a or isotype Ab treatment. (**F**) Experimental schema outlines the ACT of TdTomato^+^ WT or KO Tregs and/or CD8^+^ T cells into *Tcrbd^–/–^* mice (recipient) following MC38 tumor cell implantation (0.6 × 10^6^ cells, s.c.). TdTomato^+^ WT or gp96-KO Tregs from SPLs of TdTomato^+^ WT and TdTomato^+^ KO donor mice were isolated, preactivated, and adoptively transferred into mice bearing day-2 MC38 tumors. In parallel, CD8^+^ T cells were isolated from dLNs of C57BL/6 mice bearing day-12 MC38 tumors, stimulated in vitro for 3 days, and transferred into recipient mice on day 4. (**G**–**I**) MC38 tumor growth curves among the indicated 5 groups of mice (*n* = 4–7/group). Results are representative of 3 independent experiments. Data are shown as the mean ± SEM. **P < 0.01, ***P < 0.001, and ****P < 0.0001 (KO vs. WT), by 2-tailed Student’s *t* test for comparisons of different experimental groups where multiple comparisons were not performed (**B** and **C**) and repeated-measures 2-way ANOVA for analysis of tumor growth curves (**E**, **H** and **I**).

So far, we demonstrated that deleting gp96 from Tregs resulted in 2 major defects. First, gp96-null Tregs cannot migrate into the TME due to dysfunctional LFA-1 expression. Second, they cannot convert to effector Tregs, which is associated with suboptimal IL-2 signaling. Given this unique phenotype, our Treg-specific gp96 animal model presents an ideal loss-of-function approach to study the effect of intratumoral Tregs on antitumor CD8^+^ T cell immunity. Spectral flow cytometric analysis of CD45^+^ TILs in MC38 tumors revealed a striking and consistent increase in CD8^+^ TILs in KO mice, reaching over 40% of CD45^+^ TILs by day 14, which was over 4-fold more abundant than that seen in WT tumors (Figure 6, A and B). Additionally, CD8^+^ TILs showed increased activation during the MC38 progression in KO mice (Figure 6C). These CD8^+^ TILs were required for tumor control, as depletion of CD8^+^ T cells restored tumor growth (Figure 6, D and E, and Supplemental Table 5). To explicitly evaluate the effect of Tregs and CD8^+^ T cells on tumor control, we performed an adoptive cell transfer experiment using *Tcrbd^–/–^* recipient mice (Figure 6F). Without CD8^+^ T cell cotransfer, MC38 tumor growth was comparable after receiving TdTomato^+^ WT Tregs alone (group 1), TdTomato^+^ KO Tregs alone (group 2), or no adoptive cell transfer (ACT) (group 3) (Figure 6G and Supplemental Table 5). Tumors in group 1 grew slightly more quickly than did those in group 4, which received both TdTomato^+^ WT Tregs and CD8^+^ T cells (group 4), without reaching statistical significance (Figure 6H and Supplemental Table 5). However, when TdTomato^+^ KO Tregs were cotransferred with WT CD8^+^ T cells (group 5), we observed significantly better tumor control compared with transfer of KO Tregs alone (group 2) (Figure 6I and Supplemental Table 5). These data suggest that tumor rejection by gp96 Treg–KO mice required CD8^+^ T cells.

**Figure 7:**
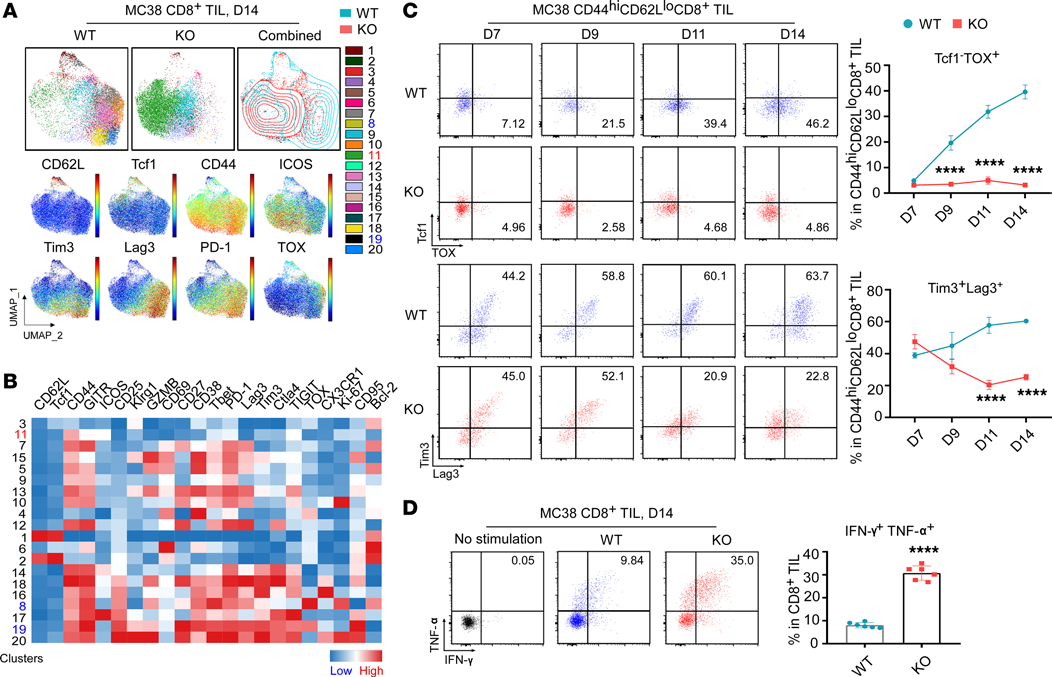
Upon gp96 deletion, the lack of infiltrating Tregs inhibits TOX-mediated CD8^+^ TIL exhaustion in MC38 tumors. (**A**) Spectral flow cytometric analysis of CD8^+^ TILs collected from day-14 MC38 tumors grown in WT and KO mice (*n* = 7/group). Cluster 11 (CD44^hi^CD62L^lo^ICOS^+^CD8^+^ Teffs; highlighted in red) increased in the KO group; clusters 8 and 19 (CD44^hi^CD62L^lo^Tcf1^–^TOX^+^PD-1^+^Lag3^+^Tim3^+^CD8^+^ subsets; highlighted in blue) decreased in the KO group. Expression distribution of the indicated markers is shown in the bottom plots. (**B**) Heatmap of marker expression by cluster. (**C**) Representative flow cytometric plots and summary graphs compare the percentages of the Tcf1^–^TOX^+^ and Tim3^+^Lag3^+^ population within CD44^hi^CD62L^lo^CD8^+^ TILs from day-7, -9, -11, and -14 MC38 tumors grown in WT and KO mice pretreated with tamoxifen (days –10 to 0; *n* = 5–8/group). (**D**) Representative flow cytometric plots and summary graph indicating the percentages of IFN-γ^+^TNF-α^+^ CD8^+^ TILs from day-14 MC38 tumors grown in WT and KO mice (*n* = 6/group) after a 5-hour ex vivo TCR stimulation with anti-CD3 and anti-CD28 Abs. Results are representative of more than 3 independent experiments. Data are shown as the mean ± SEM. ****P < 0.0001 (KO vs. WT), by 2-tailed Student’s *t* test for comparisons of different experimental groups (**C** and **D**).

Next, we performed phenotypic profiling of CD8^+^ TILs from tumors grown in WT and KO mice (Figure 7, A and B). During the early phase of tumor growth (days 7–9), gp96 deletion induced a loss of Treg intratumoral infiltration and significantly augmented the activity of CD8^+^ TILs, as evidenced by increased CD44^hi^CD62^lo^, GZMB^+^Tcf-1^–^, ICOS^+^Tcf-1^–^ effector cell populations and a Ki-67^+^ proliferating subset (Figure 6C and Supplemental Figure 12, A–F). During the later phase of tumor growth (days 11–14) (Figure 7C and Supplemental Figure 12, G–I), CD44^hi^CD62^lo^Tcf1^–^CD8^+^ TILs in WT mice gradually gained expression of immune checkpoint molecules (PD-1, Tim3, Lag3, Ctla4) and TOX, a master transcription factor responsible for reprograming CD8^+^ T cells into the exhausted state (35, 36). TOX is specifically increased in dysfunctional CD8^+^ T cells during tumor progression or chronic viral infection and is critical for controlling the expression of coinhibitory receptors during persistent TCR stimulation (37). In stark contrast, there was no TOX induction in the CD8^+^ TILs from KO mice (Figure 7C, upper panels, and Supplemental Figure 12, G–I). Importantly, we detected comparable expression levels of Lag3, PD-1, and Tim3 in CD44^hi^CD62^lo^Tcf1^–^ CD8^+^ TILs between WT and KO mice at early time points (day 7), but CD8^+^ TILs from KO mice showed no further upregulation of expression over time (Figure 7C, lower panels, and Supplemental Figure 12, G–I). These findings strongly suggest that intratumoral Tregs in WT tumors promoted CD8^+^ TIL dysfunction by reinforcing TOX induction in TILs and that Teffs could fully differentiate in the absence of Tregs. Indeed, upon acute ex vivo TCR activation, CD8^+^ TILs from KO mice produced more than 3-fold higher levels of cytokines, including IFN-γ and TNF-α, compared with WT mice (Figure 7D). Taken together, we conclude that loss of the gp96/LFA-1 axis resulted in fewer TME-infiltrating Tregs, which boosted the effector activity of antitumor CD8^+^ TILs and prevented TOX-dependent CD8^+^ T cell exhaustion.

**Figure 8:**
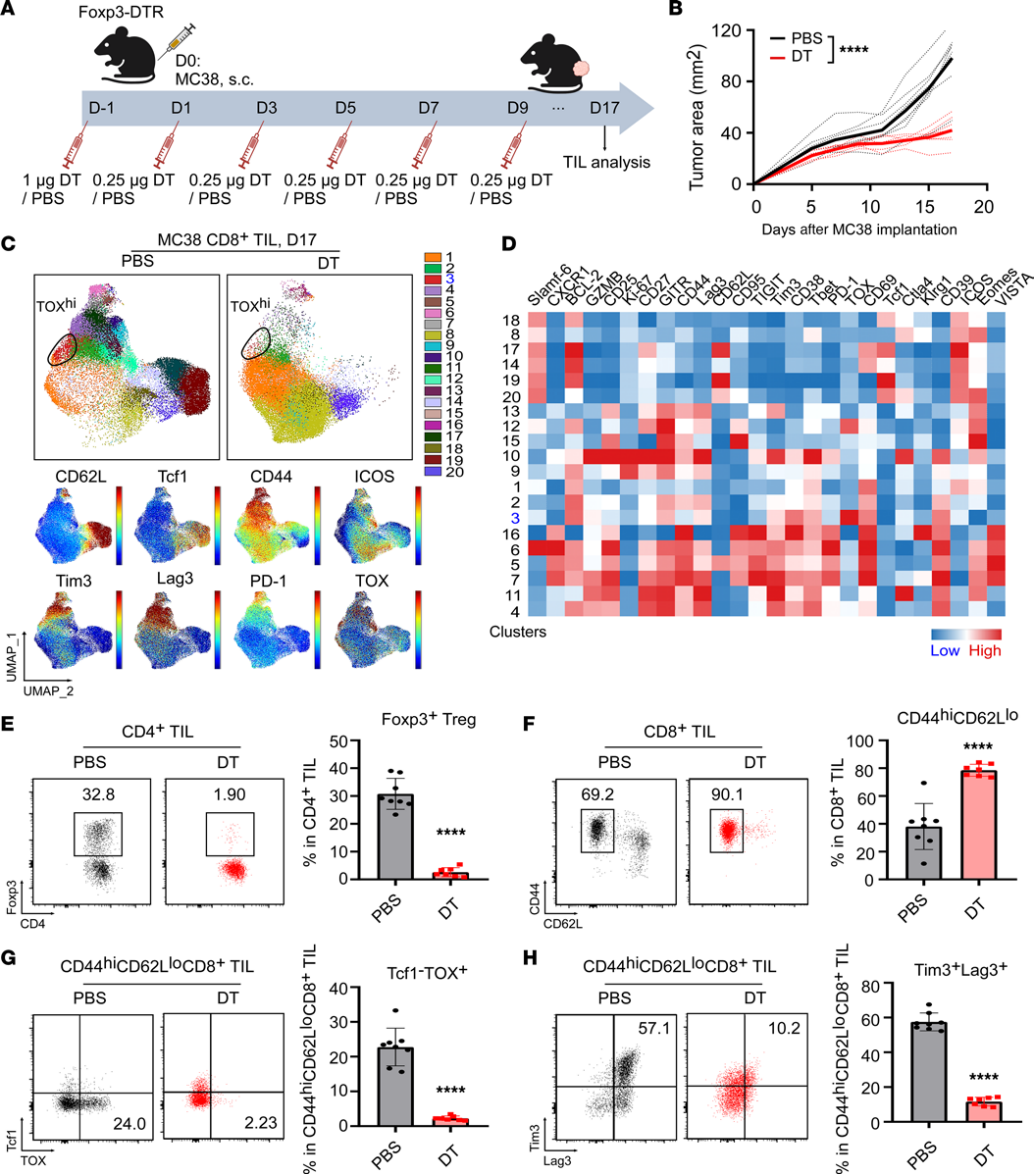
Depletion of Tregs attenuates TOX-mediated CD8^+^ TIL exhaustion in MC38 tumors from Foxp3^DTR^ mice. (**A**) Experimental schema illustrates the process of DT or PBS treatment in Foxp3^DTR^ mice (*n* = 7–8/group) implanted with MC38 tumor cells (day 0; 2 × 10^6^ MC38 cells/mouse). (**B**) Tumor growth curves of MC38 tumor cells grown in Foxp3^DTR^ mice receiving DT or PBS treatment. (**C**) Spectral flow cytometric analysis of CD8^+^ TILs collected from day-17 MC38 tumors grown in Foxp3^DTR^ mice. Cluster 3 (CD44^hi^CD62L^lo^Tcf1^–^TOX^+^PD-1^+^Lag3^+^Tim3^+^CD8^+^ subset; highlighted in blue) decreased in the DT group. Expression distribution of the indicated markers is shown in the bottom plots. (**A**) Heatmap visualization of marker expression by cluster. (**E**–**H**) Representative flow cytometric plots and summary graph indicate the percentages of Foxp3^+^ Tregs (within CD4^+^ TILs) (**E**), the CD44^hi^CD62L^lo^ population (within CD8^+^ TILs) (**F**), the Tcf1^–^TOX^+^ subset (within CD44^hi^CD62L^lo^CD8^+^ TILs) (**G**), and Tim3^+^Lag3^+^ cells (within CD44^hi^CD62L^lo^CD8^+^ TILs) (**H**) among groups. Results are representative of 3 independent experiments. Data are shown as the mean ± SEM. ****P < 0.0001 (DT vs. PBS), by repeated-measures 2-way ANOVA for tumor growth curves (**B**) and 2-tailed Student’s *t* test for comparisons of different experimental groups (**E**–**H**).

To further confirm the effect of Tregs on TOX-associated CD8^+^ TIL exhaustion, we applied a Treg-specific depletion strategy using Foxp3^DTR^ mice, in which administration of diphtheria toxin (DT) ablated all Tregs. The mice were inoculated with MC38 tumors on day 0 and treated with either DT or PBS at the indicated time points (Figure 8A). As expected, mice treated with DT had improved eradication of MC38 tumors (Figure 8B and Supplemental Table 6). Unlike Treg-specific deletion of gp96, the tumor eradication was not complete in this model, which could be due to differences in the experimental setting or perhaps to gaining some effector function by gp96-KO Tregs. Spectral flow cytometric analysis of day-17 MC38 CD8^+^ TILs revealed a substantial reduction in cluster 3 (CD44^hi^CD62L^lo^Tcf1^–^TOX^+^PD-1^+^Lag3^+^Tim3^+^CD8^+^ exhausted subset) in the DT-treated mice (Figure 8, C and D). Treg depletion by DT (Figure 8E) promoted CD8^+^ TIL activation, as evidenced by the increased frequency of the CD44^hi^CD62^lo^ TIL subset (Figure 8F). Finally, Treg depletion inhibited CD8^+^ TIL expression of TOX and inhibitory receptors, including Lag3 and Tim3 (Figure 8, G and H), which highlights the crucial role of Tregs in fostering the development or maintenance of exhausted CD8^+^ TILs, consistent with our findings in gp96 Treg–KO models.

To provide clinical context, we analyzed RNA-Seq data from TCGA database, including data on bladder, breast, colon, head and neck, kidney, lung, pancreatic, and skin cancer patient cohorts. We found a strong positive correlation between the Treg signature (FOXP3) and T cell exhaustion signatures (TOX, HAVCR2, PDCD1, TIGIT, LAG3, CTLA4, and CXCL13) in various treatment-naive human cancers (Supplemental Figure 13), indicating that elevated Treg numbers in the TME, probably promote CD8^+^ T cell exhaustion in human cancers.

### Sequestration of IL-2 underlies the mechanisms of Tregs to promote TOX expression and CD8^+^ T cell exhaustion.

**Figure 9:**
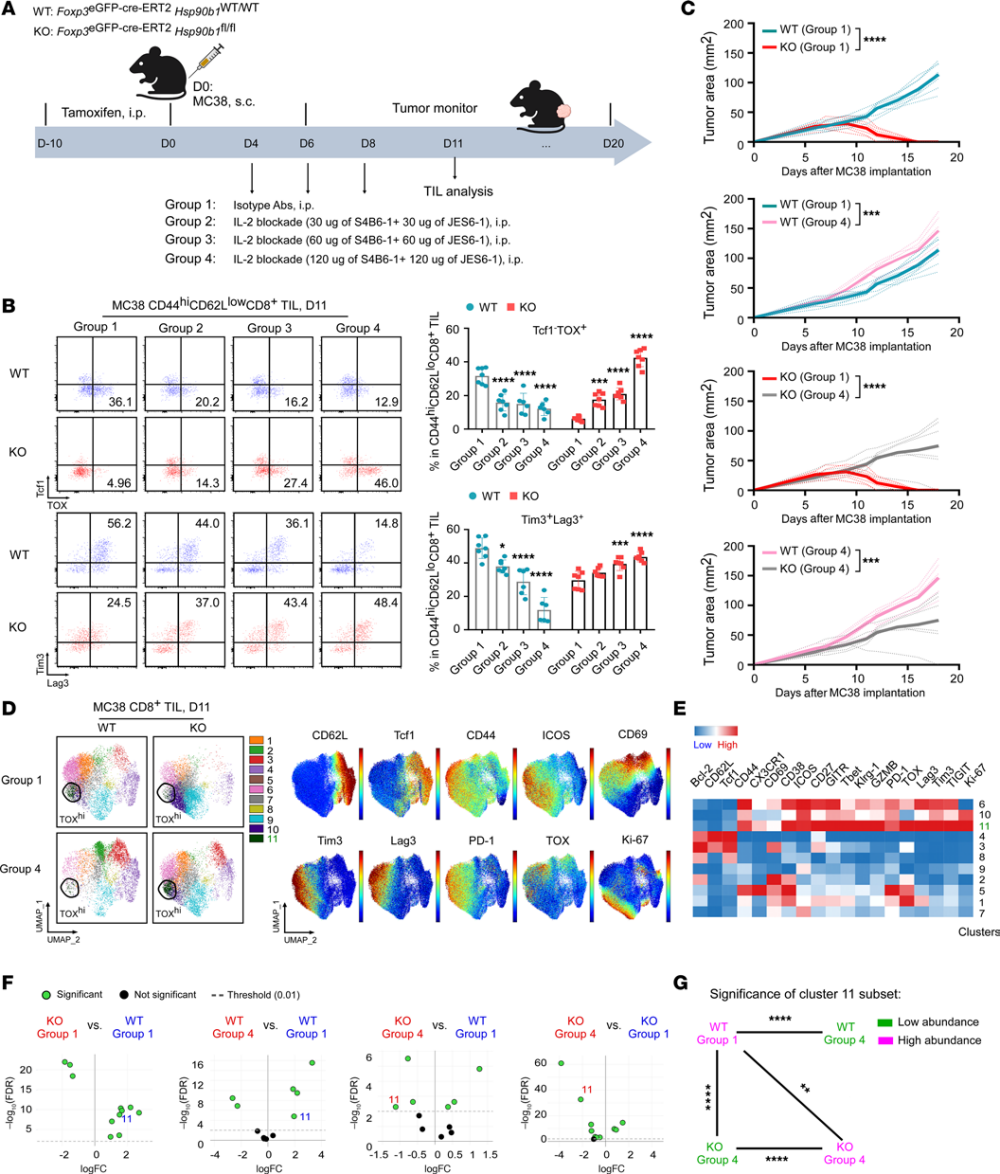
In the absence of Tregs, IL-2 blockade induces TOX-mediated CD8^+^ TIL exhaustion, promoting MC38 tumor growth. (**A**) Experimental schema depicts the administration of IL-2–blocking Abs (S4B6-1 and JES6-1) or matched isotype Abs in MC38 tumor–bearing WT and KO mice (2 × 10^6^ MC38 cells/mouse, s.c.; *n* = 6–8/group). (**B**) Representative flow cytometric plots and summary graphs show the frequencies of Tcf1^–^TOX^+^ and Tim3^+^Lag3^+^ cells within CD44^hi^CD62L^lo^CD8^+^ TILs from day-11 MC38 tumors grown in WT and KO mice (*n* = 6–7/group). (**C**) Growth curves of MC38 tumors grown in WT and KO mice that received IL-2 blockade (group 4; 120 μg for each Ab) or isotype Ab treatment (group 1). Tumor sizes were measured. (**D**) Spectral flow cytometric analysis of CD8^+^ TILs isolated from day-11 MC38 tumors grown in WT and KO mice treated with IL-2 blockade (group 4) or isotype Ab (group 1). Cluster 11 (CD44^hi^CD62L^lo^Tcf1^–^TOX^+^PD-1^+^Lag3^+^Tim3^+^CD8^+^ subset), shown as circles, is highlighted in green. Expression distribution of the indicated markers is also shown in the plots on the right. (**E**) Heatmap of the indicated markers by cluster. (**F**) edgeR analysis indicates significant differences in CD8^+^ TIL clusters between KO and WT groups (cluster 11 is highlighted). (**G**) Significant differences in cluster 11 enrichment among the indicated groups. Green indicates low abundance of cluster 11; purple indicates high abundance of cluster 11. Results are representative of 3 independent experiments. Data are shown as the mean ± SEM. *P < 0.05, **P < 0.01, ***P < 0.001, and ****P < 0.0001 (IL-2 blockade vs. isotype). A 2-tailed Student’s *t* test was performed for comparisons of different experimental groups (**G**) where multiple comparisons were not performed; for analyses where multiple comparisons were performed, a 1-way ANOVA with Šidák’s multiple-comparison correction was performed for comparison of treatment groups across genotypes (**B**); a 1-way ANOVA analysis with Dunnett’s multiple-comparison correction was performed for comparison within groups for treatments compared with the respective isotype (**B**). Tumor growth were curves were analyzed by repeated-measures 2-way ANOVA (**C**).

Finally, we investigated the mechanism by which TOX is downregulated in CD8^+^ TILs following Treg-specific gp96 deletion. Tregs can sequester intratumoral IL-2 via high-affinity IL-2 receptors, including CD25, CD122, and CD132 (1, 19); this mechanism mediates much of the suppressive capacity of these Tregs against Teffs (38–41). Liu et al. demonstrated that IL-2 in the TME plays roles in both the activation of CD8^+^ T cells and the induction of CD8^+^ T cell exhaustion through the hydroxytryptophan/AhR (5-HTP/AhR) pathway (42). Beltra et al. demonstrated the role of the IL-2/STAT5 axis in epigenetic “rewiring” of exhausted CD8^+^ T cells toward a more functional state (43). However, to what extent Tregs consume or sequester intratumoral IL-2 and regulate TOX-dependent T cell dysfunction remains unknown. Therefore, we treated WT and KO tumor–bearing mice with IL-2–blocking Abs (clones S4B6-1 and JES6-1) (Figure 9A and Supplemental Figure 14A). In KO mice, we found that early IL-2 blockade (days 4–8) was sufficient to restore the expression of TOX and inhibitory receptors on CD8^+^ TILs from later-stage (day-11) MC38 tumors (Figure 9B). IL-2 blockade also attenuated CD8^+^ TIL activation, as determined by a reduction of the CD44^hi^CD62^lo^ subset in both WT and gp96-KO mice (Supplemental Figure 14B). Interestingly, even treated with a high dose (120 μg) of IL-2–blocking Abs, KO mice lacking tumor-infiltrating Tregs displayed a relatively high frequency (~79%) of CD44^hi^CD62^lo^ CD8^+^ TILs, comparable to that in WT mice without IL-2 blockade (Supplemental Figure 14B). This implies that, without Tregs, low levels of IL-2 in the TME can induce TOX expression in CD8^+^ TILs without hindering their activation. In contrast, in WT mice with abundant intratumoral Tregs, IL-2 blockade markedly inhibited CD8^+^ TIL activation and attenuated the expression of TOX and immune checkpoint molecules (Figure 9B and Supplemental Figure 14B), consistent with previous observations (42). These findings suggested that the induction of TOX and coinhibitory receptors in CD8^+^ TILs depended on both Tregs and suboptimal levels of IL-2. Unsurprisingly, when given a high dose (120 μg) of IL-2–blocking Abs, gp96-KO mice did not show rejection of MC38 growth (Figure 9C and Supplemental Table 7). Collectively, these findings underline a dynamic and nuanced role for Tregs and IL-2 signaling in regulating CD8^+^ TIL TOX expression. IL-2 depletion enriched an exhaustion-prone subset of CD8^+^ TILs (cluster 11), characterized by CD44^hi^Tcf1^–^TOX^+^Tim3^+^Lag3^+^PD-1^+^TIGIT^+^CD8^+^ in MC38 tumor–bearing KO mice (Figure 9, D–G). Treatment of MC38 tumor–bearing mice with exogenous IL-2 and anti–IL-2 complex (clone S4B6-1), which is known to potently expand CD8^+^ T cells (32, 44, 45), significantly reduced TOX^hi^ cluster 11 TILs (Supplemental Figure 14, C and E). The activated CD44^hi^CD62^lo^ CD8^+^ TIL population was slightly decreased by IL-2 and S4B6-1 administration (Supplemental Figure 14B), consistent with progressive tumor growth (Supplemental Figure 14F and Supplemental Table 7). Collectively, we concluded that infiltrating Tregs controlled by the gp96/LFA-1 axis reinforced TOX expression and promoted CD8^+^ TIL exhaustion in part through competition for IL-2 in the TME.

## Discussion

During the past decade, ICB treatments such as anti-CTLA4 and anti–PD-1 Abs have revolutionized the treatment of human malignancies (46–48). Notably, these Abs exhibit unprecedented antitumor activity in part by blocking Treg-mediated immunosuppression (49). Depleting or functionally modulating the suppressive function of Tregs can shift the balance from immune evasion to immune activation and is thus considered one of the cornerstones of antitumor immunity (50). There are multiple ways to target Tregs for cancer immunotherapies, including utilizing Abs against Treg activation molecules, such as CD25, CTLA4, and OX40, and strategies to block the chemokine receptor CCR4 (37, 51–57). However, targeting CD25 may also dampen Teff responses due to shared CD25 expression of CD25 (58). The most concerning obstacle associated with Treg-targeted therapy is the risk of inducing serious or fatal autoimmunity. Selective inhibition of Tregs in tumors but not in the secondary lymphoid organs may enhance cancer immunotherapy without eliciting deleterious autoimmunity. However, this promise remains unrealized. In this study, we discovered that deletion of gp96 from committed Tregs in mice resulted in complete blockade of LFA-1–dependent infiltration of Tregs into the TME and compromised the activation of effector Tregs, leading to enhanced CD8^+^ TIL activation, hindrance of IL-2–dependent, TOX-mediated T cell exhaustion, and a superior ability to eradicate multiple tumor types without inducing autoimmunity.

gp96 is an essential molecular chaperone for various proteins, including TLRs, the platelet glycoprotein Ib/IX/V complexes, GARP, the Wnt coreceptor LRP6, and integrins (12, 59, 60). Integrins are surface receptors that regulate T cell adhesion, activation, and migration. Among the diverse group of leukocyte-specific integrins, LFA-1 is a key player in T cell biology, as it is highly expressed on T cells and mediates tissue-specific trafficking (61, 62). LFA-1 is composed of α chain CD11a (αL) and a shared β subunit CD18 (β2). Via binding with its ligand intracellular adhesion receptor 1 (ICAM1), LFA-1 helps T cells adhere to the endothelium, communicate with other inflammatory cells, and elicit protective immunity. Marski et al. reported that deletion of CD18 in Tregs can lead to diminished population sizes with impaired suppressive function (20, 63). However, the contribution of LFA-1 to Treg homing and TME trafficking has never to our knowledge been reported. Our current work demonstrated that LFA-1 was indispensable for Treg TME infiltration. Via CRISPR/Cas9, we found that αV, αE, β1, β3, or β7 integrins were not required by Tregs for TME infiltration, emphasizing the preference of LFA-1 over other integrins. TCGA analysis further supported the predominance of LFA-1 expression in tumor-infiltrating Tregs, particularly in breast cancer, correlating with a poor prognosis across various other cancer types. Notably, both animal work and human data revealed a minimal effect of LFA-1 on the tumor-specific trafficking of other CD4^+^ T cell subsets, indicating specificity in mediating Treg recruitment. Previous studies have addressed the importance of LFA-1 in regulating the activation rather than the infiltration of CD8^+^ T cells and NK cells into tumors (23–25). The specific involvement of LFA-1 in intratumoral Treg trafficking highlights the heterogeneity of the immune landscape and the distinct migratory behavior of diverse immune cells within tumors.

While the absence of Tregs in the TME due to gp96 deletion is a major contributor to tumor regression, the comprehensive analysis of functional alterations in gp96-null Tregs remained an essential aspect of our investigation. gp96 deletion leads to a notable downregulation of CD25, Foxp3, and other activation markers. However, exogenous IL-2 could restore the observed phenotypic changes of gp96-KO Tregs, suggesting that the defect in gp96-null Tregs may arise from reduced IL-2 availability rather than intrinsic signaling abnormalities. Brockdorff et al. reported that IL-2 promotes some aspects of signaling in an integrin-dependent manner (34). Integrin activation has been shown to induce CD25 expression (33, 63), and β2 integrin plays a role in Th2 but not Th1 infiltration to sites of inflammation (64). Thus, the suboptimal IL-2/STAT5 signaling in gp96-KO Tregs is consistent with the positive crosstalk between integrin and IL-2 signaling. Collectively, targeting Treg-specific gp96 not only prevents Treg infiltration into the TME but also hampers their activation due to suboptimal IL-2/p-STAT5 signaling, contributing to heightened antitumor immunity and superior tumor control.

Our present work further sheds light on how Treg infiltration into solid tumors effects on CD8^+^ T cell exhaustion. Tregs, known for their highly suppressive and hyperproliferative features in the TME, express heightened levels of CD25, CTLA4, GITR, OX40, ICOS, and neuropilin-1 and accumulate significantly during tumor development (4, 50, 65–70). While Tregs tend to exert immunosuppressive effects, CD8^+^ TILs, distinguished by their cytotoxicity function, serve as potent effectors against tumors (71). Our findings revealed that the absence of Tregs allowed for increased accumulation and activation of CD8^+^ TILs, which directly controlled tumors as confirmed by the CD8^+^ T cell depletion study. However, how WT Tregs promote CD8^+^ T cell exhaustion in the TME remains incompletely understood. Sawant et al. previously reported that expression of the cytokines IL-10 and IL-35 by intratumoral Tregs cooperatively promotes BLIMP1-dependent CD8^+^ T cell exhaustion and inhibits effective antitumor immunity (72). In the current study, we demonstrate that the loss of infiltrating Tregs not only enhanced early-stage CD8^+^ TIL effector activity but also inhibited TOX induction, preventing the CD4^+^ T cell transition into functional exhaustion during tumor progression. This was reinforced by a Treg-specific depletion mouse model using Foxp3^DTR^ mice, underscoring the requirement of Tregs in mediating TOX-dependent CD8^+^ TIL exhaustion. Our findings resonate with a recent study which showed that targeting CCR8^+^ Tregs with an anti-CCR8 Ab increases CD8^+^ effector cells and decreases CD8^+^ TOX^hi^ exhausted cells, leading to potent antitumor effects (73). A comprehensive analysis of TCGA data further revealed a strong positive correlation between Treg infiltration and CD8^+^ T cell exhaustion across diverse human cancers, suggesting a potential role for Tregs in transcriptionally inducing TOX and other signatures, ultimately promoting cancer progression.

It remains plausible that TOX expression is necessary for CD8^+^ T cells to overcome Treg-mediated suppression and ensure survival in the Treg-replete TME, given the known roles of TOX as a prosurvival factor (36, 74). In a Treg-depleted TME, such as when gp96 or LFA1 was conditionally deleted, TOX expression by CD8^+^ T cells became unnecessary. Undoubtedly, it will be fruitful to conduct future studies to gain an understanding of how Tregs induce TOX in the CD8^+^ T cell compartment and the consequences on cancer immunity and therapeutic responsiveness.

Finally, our kinetics study revealed, for the first time to our knowledge, that by sequestering and consuming IL-2, intratumoral Tregs hindered CD8^+^ TIL hyperactivation in the early phase of tumor growth and promoted TOX-dependent CD8^+^ TIL exhaustion during tumor progression. Although IL-2 has been associated with promoting the expression of coinhibitory receptors such as TIM3 and PD-1 (42, 75, 76), its effect on CD8^+^ T cell exhaustion remains unclear. Recent research suggested that IL-2–based treatment using a *cis*-targeted CD8–IL-2 fusion protein could rescue dysfunctional hepatitis B virus–specific CD8^+^ T cells by increasing their IFN-γ and granzyme B production (77). In addition, a PD-1 *cis*-targeted IL-2Rβγ agonist induced antigen-specific CD8^+^ T cell states with better effector potential, deviating from T cell exhaustion (78). In the present study, we found that TOX induction and CD8^+^ T cell exhaustion could be reinduced by IL-2 blockade even in Treg-specific gp96-KO mice. We also showed that exogenous IL-2 attenuated TOX-mediated CD8^+^ T cell exhaustion in WT mice. This unveils 2 key insights into CD8^+^ T cell exhaustion within the TME. First, Tregs promoted CD8^+^ T cell exhaustion in the tumor by being the main consumer of IL-2; restoration of IL-2 levels locally by blocking gp96/LFA-1–dependent tumor infiltration of Treg was able to reverse CD8^+^ T cell exhaustion. Second, IL-2 dually tuned TOX expression by CD8^+^ TILs; either excessive or inadequate IL-2 stimulation alone failed to induce TOX expression. We believe that increasing IL-2 bioavailability locally can effectively block TOX induction in CD8^+^ T cells and thus curtail their programming into an exhausted state in the TME, consistent with a recent finding (79). More studies are needed to deepen our understanding of the molecular mechanisms by which Tregs promote TOX-mediated CD8^+^ TIL exhaustion. In addition to hijacking IL-2, Tregs may also induce T cell tolerance by downregulating MHCs from antigen-presenting cells (80) and secreting potent immune-suppressive cytokines such as TGF-β (11, 81, 82), among many other mechanisms of immune suppression.

In conclusion, our study has uncovered 2 critical aspects of Treg biology in cancer immunity (Supplemental Figure 10). First, targeting the gp96/LFA-1 axis in Tregs could effectively deplete intratumoral Tregs without disturbing immune homeostasis. Second, limiting Treg infiltration into the TME prevented CD8^+^ TILs from gaining TOX and functional exhaustion in an IL-2–dependent manner, leading to effective eradication of cancer. Selective silencing of gp96 and/or LFA-1 in Tregs might prove to be an effective strategy for cancer immunotherapy in the future.

## Methods

### Sex as a biological variable.

Our study examined male and female animals, and similar findings are reported for both sexes.

### Mice.

All mice experiments were performed using age- and sex-matched (8- to 12-week-old) mice. See Supplemental Methods for breeding and mouse treatment details.

### Tumor models.

WT and KO mice were inoculated s.c. in the right flank with 2 × 10^6^ MC38, 1 × 10^6^ MB49, and 2.5 × 10^5^ B16-F10 tumor cells in 100 μL PBS on day 0. For tumor rechallenge, all tumor-regressed KO mice along with age-matched, tumor-naive WT mice were rechallenged s.c. in the opposite flank with 2 × 10^6^ MC38, 1 × 10^6^ MB49, or 2.5 × 10^5^ B16-F10 tumor cells on day 60 after primary tumor cell inoculation. See Supplemental Methods for details on the cell lines and in vivo depletion experiments.

### Flow cytometry.

TILs were prepared as previously described (83). The fluorochrome-conjugated Abs against mouse antigens in the indicated flow cytometry panels were utilized (see Supplemental Methods). All flow samples were acquired using Cytek Aurora, and results were analyzed with FlowJo VX software (Tree Star) or OMIQ Flow Cytometry software (Dotmatics). We used uniform manifold approximation and projection (UMAP) for the visualization of spectral flow cytometry.

### iTreg differentiation and CRISPR/Cas9 electroporation.

Naive CD4^+^ T cells were freshly isolated from SPLs of JAX-WT (The Jackson Laboratory) mice using the Mouse Naive CD4^+^ T Cell Isolation Kit (Miltenyi Biotec) according to the manufacturer’s protocol. Cells were stimulated under Treg-skewed conditions for 5 days. On day 2, CRISPR/Cas9 electroporation was performed to delete the indicated integrins in iTregs using the Lonza 4D-Nucleofector System (84, 85). Detailed guide RNA (gRNA sequences in Supplemental Table 8. The KO efficiency was determined on day 5 by flow cytometry. See Supplemental Methods for details.

### In vitro Treg suppression assay.

Murine SPL–derived nonregulatory CD4^+^ Teffs and CD8^+^ T cells were used as responder T cells. Splenic Tregs (suppressors) were purified by FACS based on fluorescence tdTomato protein from WT-tdTomato or KO-tdTomato mice. Cocultures were set up with either 5 × 10^4^ CD4^+^ Teffs or 1 × 10^4^ CD8^+^ T cells along with WT or KO Tregs at the indicated ratios. Cell proliferation of responder CD4^+^ Teffs or CD8^+^ T cells was determined by flow cytometry on the basis of their dilutions of CTV fluorescence intensity after stimulation for the designated durations. See Supplemental Methods for details.

### Adoptive transfer model.

For adoptive transfer of integrin-deficient iTreg experiments, nucleofected iTregs (2 × 10^6^) containing specific targeting sgRNAs or nontargeting control sgRNAs along with CAS9 were i.v. injected via the tail into *Rag2^–/–^* recipient mice on day 3 after MC38 tumor cell implantation. On day 10, the mice were sacrificed and tumors and SPLs harvested for Treg ex vivo analysis.

For adoptive transfer of WT and gp96-null Tregs, splenic CD4^+^ T cells were enriched using the EasySep Mouse CD4^+^ T Cell Isolation Kit (STEMCELL Technologies), and thereafter, tdTomato^+^ Tregs were isolated by FACS from SPLs of TdTomato-WT and TdTomato-KO mice. Isolated Tregs were subsequently activated and expanded in vitro using a mouse Treg Expansion Kit (Miltenyi Biotec) together with 2,000 U/mL rhIL-2 (R&D Systems) for 3 days. Preactivated tdTomato^+^ WT or gp96-KO Tregs (2 × 10^6^ per mouse) were adoptively transferred i.v. via the tail into *Rag2^–/–^* recipient mice on day 3 after MC38 inoculation. On day 10, Treg accumulation in the SPL and the tumor was determined by flow cytometry.

For cotransfer of Tregs and CD8^+^ T cells, tdTomato^+^ WT or gp96-KO Tregs were isolated and preactivated as mentioned above and adoptively transferred into MC38-bearing *Tcrbd^–/–^* recipient mice 2 days after tumor cell implantation. In parallel, CD8^+^ T cells were isolated by FACS from dLNs of day-12 MC38-bearing JAX-WT mice and stimulated with 5 μg/mL plate-bound anti-CD3 Ab (clone 17A2, BioLegend, catalog 100202), 2.5 μg/mL soluble anti-CD28 Ab (clone 37.51, BioLegend, catalog 102121), and 100 IU/mL rhIL-2 (R&D Systems) for 3 days. Donor CD8^+^ T cells were i.v. transferred into MC38-bearing *Tcrbd^–/–^* recipient mice on day 4 after tumor cell implantation. Tumor growth in *Tcrbd^–/–^* recipient mice was monitored at the indicated time points.

### RNA-Seq and gene correlation.

Splenic GFP^+^ Tregs (1 × 10^6^) were purified from WT (*n* = 4) and KO mice (*n* = 4) via FACS isolation. RNA was extracted using the RNeasy Micro Kit (QIAGEN) following the manufacturer’s standard protocol. For RNA-Seq, libraries were prepared using the NEBNext Ultra RNA Library Prep Kit for Illumina (New England BioLabs [NEB]) following the manufacturer’s recommendations, with index codes. Additional RNA-Seq and gene correlation details are included in Supplemental Methods.

### Statistics.

Except for RNA-Seq analysis, statistical significance was determined using GraphPad Prism 9.0 (GraphPad Software). Tumor growth curves and body weight curves were analyzed using repeated-measures 2-way ANOVA. Survival incidence analysis was performed by log-rank (Mantel-Cox) test. For comparison of a single variable between 2 groups, a 2-tailed Student’s *t* test was applied. A *P* value of less than 0.05 was considered statistically significant. For analyses across multiple groups where variance did not significantly differ across groups, 1-way ANOVA with Dunnett’s (for comparisons across selected groups versus control), Tukey’s (for comparisons across all groups), or Šidák’s (for comparisons between preselected pairs of columns) multiple-comparison corrections was used. For analyses across multiple groups where variance significantly differed across groups, Brown-Forsyth/Welch ANOVAs with Dunnett’s T3 multiple-comparison corrections was used.

### Data availability.

The code for analysis of the bulk RNA-Seq data, the normalized gene expression matrix, and the differentially expressed gene (DEG) list for figure 5a and 5d can be accessed at https://zenodo.org/doi/10.5281/zenodo.11087687 (published April 29, 2024, version v1). The raw and processed sequencing data are deposited in the NCBI Gene Expression Omnibus (GEO) database (GEO GSE266362). All values underlying the data presented in the graphs and as means are available in the Supporting Data Values file.

### Study approval.

All mouse studies were conducted under protocols approved by the IACUC of The Ohio State University (no. 2019A00000075-R1).

## Author contributions

ZL conceived the project. LZ and ZL designed experiments and wrote the manuscript. LZ performed most experiments and conducted the related analyses. MV and NJS developed spectral flow panels for Tregs and CD8^+^ T cells, respectively. YW performed the Foxp3^DTR^ in vivo experiment. JKM performed bioinformatics analyses and contributed to manuscript revisions. WL performed iTreg differentiation experiment and helped with the in vivo tumor rejection study. HK, GX, and MPR contributed to experimental design and provided technical support. YC, QM, and TX assisted with RNA-Seq analysis and the gene correlation study. CB helped with manuscript preparation and revision. MV, MPR, FH, and HW provided critical discussion of data and assistance with project direction. All authors reviewed and approved the manuscript.

## Supplementary Material

Supplemental data

Supplemental data set 1

Supporting data values

## Version 1 05/24/2024

In-Press Preview

## Version 2 07/15/2024

Electronic publication
