# Supplementary material for: Spatial and functional targeting of intratumoral Tregs reverses CD8+ T cell exhaustion and promotes cancer immunotherapy
Source: J Clin Invest. 2024 May 23;134(14):e180080. doi: 10.1172/JCI180080 (PMC11245154; doi:10.1172/JCI180080)
Supplement: Supplemental data [file jci-134-180080-s131.pdf]

Supplemental Information

Extended Methods

Cell lines and Tumor Models:

The MC38 colon adenocarcinoma cells (American Type Culture Collection, ATCC) and MB49 bladder cancer cells (a kind gift from Dr. Laura Kasman, Medical University of South Carolina) were cultured with Dulbecco's modified Eagle's medium (DMEM) (HyClone, USA) supplemented with 10% heat-inactivated fetal bovine serum (FBS) and 1% penicillin-streptomycin. The B16-F10 melanoma cells were obtained from ATCC and maintained with RPMI 1640 media (Gibco, USA) supplemented with 10% heat-inactivated FBS and 1% penicillin-streptomycin.

WT or KO mice were inoculated subcutaneously (s.c.) on the right flank with $2×10^6$ MC38, $1×10^6$ MB49, and $2.5×10^5$ B16-F10 tumor cells in 100 µL PBS on D0, respectively. Tumor volumes were measured every day or every two days (length x width in mm) using a digital caliper starting on D5 post tumor implantation. For survival analysis, tumor-bearing mice were monitored daily and euthanized as non-survivors when the tumors sizes reached 16 mm in diameter. For tumor rechallenge, all tumor-regressed KO mice along with age-matched tumor-naïve WT mice were rechallenged s.c. in the opposite flank with $2×10^6$ MC38, $1×10^6$ MB49, or $2.5×10^5$ B16-F10 tumor cells 60 days (D60) after primary tumors inoculation.

Mice:

All mice were maintained in specific-pathogen free conditions with food and water by the University Laboratory Animal Resources (ULAR) at the Ohio State University. Hsp90b1$^{fl/fl}$ or *Hsp90b1*$^{wt/wt}$ mice on the C57BL/6 background were crossed with *Foxp3*$^{eGFP\text{-}Cre\text{-}ERT2}$ mice (The Jackson Laboratory, Stock number 016961) to generate *Foxp3*$^{eGFP\text{-}Cre\text{-}ERT2}$ *Hsp90b1*$^{fl/fl}$ (KO) or *Foxp3*$^{eGFP\text{-}Cre\text{-}ERT2}$ *Hsp90b1*$^{wt/wt}$ (WT) mice, respectively. The KO or WT mice were further bred to

B6.*Cg-Gt(ROSA)26Sor*$^{tm14(CAG\text{-}tdTomato)Hze}$/J mice (The Jackson Laboratory, Stock number 007914) to get *R26*$^{STOP\text{-}tdTomato}$*Foxp3*$^{eGFP\text{-}Cre\text{-}ERT2}$*Hsp90b1*$^{fl/fl}$ (TdTomato-KO) or *R26*$^{STOP\text{-}tdTomato}$*Foxp3*$^{eGFP\text{-}Cre\text{-}ERT2}$ *Hsp90b1*$^{wt/wt}$ (TdTomato-WT) mice. In these mice, Foxp3-expressing cells carry ERT2-Cre recombinase, which could delete the *loxP*-flanked STOP cassette in *ROSA26* locus and result in transcription of the red fluorescent protein variant tdTomato. C57BL/6 Wildtype (JAX-WT, Stock number 000664), *Tcrbd*$^{-/-}$ (Stock number 002122), *Rag2*$^{-/-}$ (Stock number 008449), and Foxp3$^{\text{-DTR}}$ (Stock number 016958) mice were also obtained from the Jackson Laboratory.

For tamoxifen administration, tamoxifen (Sigma-Aldrich) was reconstituted in peanut oil (Sigma-Aldrich) at 37°C by rotating until fully dissolved and kept at 4°C for short-term storage. Each mouse was injected intraperitoneally (i.p.) with 75 mg/kg tamoxifen for 10 consecutive days (from day -10 [D-10] to 0 [D0]) to induce *Hsp90b1* gene deletion in Tregs. For IL-2/JES6-1 complexes treatment, 1 µg recombinant mouse IL-2 (PeproTech) was incubated at room temperature (RT) with 5 µg JES6-1 antibody (BD Bioscience, Catalog No. 554424) for 15 min and IL-2/JES6-1 complexes were subsequently diluted in PBS for i.p. injections as previously described (1). Mice were given IL-2/JES6-1 complexes or PBS on day -8 (D-8), D-6, D-4, D-2, and D0 during 10-days tamoxifen administration. For diphtheria toxin (DT) treatment, Foxp3$^{\text{-DTR}}$ mice were given an initial i.p. dose of 1 µg DT in PBS one day (D-1) before MC38 tumor implantation (D0), followed by additional i.p. injections of 0.25 µg DT on D1, D3, 5 D5, D7, and D9 after tumor inoculation.

For *in vivo* depletion of CD8^{+} T cells, WT and KO mice were injected i.p. with 200 µg anti-mouse CD8 neutralizing antibody (Clone 53-6.7, Bio X Cell, Catalog No. BE0004-1) or 200 µg Rat IgG2a isotype control (Clone 2A3, Bio X Cell, Catalog No. BE0089) three days (D-3) prior to tumor implantation, followed by 100 µg every 3 days thereafter. For LFA-1 antibody (anti-

CD11a) treatment, JAX-WT mice were i.p. given 200 μg anti-mouse CD11a antibody (Clone M17/4, Bio X Cell, Catalog No. BE0006) or 200 μg Rat IgG2a isotype control antibody (Clone 2A3, Bio X Cell, Catalog No. BE0089) every two days starting from D4 post MC38 inoculation. For IL-2 blockade experiment, mice were i.p. injected with a cocktail of clone S4B6-1 anti-mouse IL-2 antibody (Bio X Cell, Catalog No. BE0043-1) and clone JES6-1 anti-mouse IL-2 antibody (Bio X Cell, Catalog No. BE0043) or isotype antibody (Rat IgG2a, Clone 2A3, Bio X Cell, Catalog No. BE0089), as previously described(2-4), at three different dosages (30 ug, 60 μg and 120 ug of each antibody per mouse) on D4, D6 and D8 post MC38 implantation. For IL-2/S4B6-1 complexes treatment(5-7), 1.5 μg recombinant mouse IL-2 (PeproTech) was incubated with 15 μg S4B6-1 antibody (Bio X Cell, Catalog No. BE0043-1) in PBS at RT for 15 min and were i.p. injected into mice on day D4, D6 and D8 post MC38 implantation.

Histology and cytokine analysis

WT and KO mice were given 10 days treatment of tamoxifen. 90 days (D90) later, mouse organs including the lung, liver, and gut were harvested and fixed. After embedded in paraffin, tissue sections were processed for H&E staining according to standard protocols. For cytokine analysis, serum was collected by cheek bleeding and frozen at -20°C before examination. Serum levels of IL-6, IFN-γ, and IL-10 were determined using the mouse IL-6 Quantikine ELISA Kit (R&D Systems), the IFN-γ mouse ELISA Kit (ThermoFishier Scientific), and the mouse IL-10 Quantikine ELISA Kit (R&D Systems) according to the instructions, respectively.

Flow cytometry:

Immune cells from murine SPLs and pLNs including inguinal LNs, axillary LNs, and cervical LNs were harvested by mechanic disruption through 70-mm strainers (Thermo Fisher Scientific). Red blood cells in SPLs were lysed using Red Blood Cell Lysis Buffer (BioLegend). Tumors were mechanically disrupted and subjected to digestion using 1 mg/mL Collagenase A (Roche),

2 mg/ml collagenase D (Roche), and 100 mg/ml DNase (Sigma) for 35 min at 37°C. Single-cell suspensions were collected from the flow-through passing a 40-mm strainer (Thermo Fisher Scientific). Cells were stained with LIVE/DEAD™ Fixable Blue Dead Cell Stain Kit (Invitrogen) at room temperature (RT) for 10 minutes, followed by anti-mouse CD16/32 (Clone 2.4G2, Invitrogen, Catalog No. 14-0161-82) and extracellular surface markers at 4°C for 30 minutes. Intracellular staining was performed using the Foxp3 transcription factor staining kit (Invitrogen) according to the manufacturer's instructions. ***Treg panel:*** Anti-CD45 (Clone 30-F11, Brilliant Violet 510, BioLegend, Catalog No. 103138), anti-CD3 (Clone 17A2, BUV737, BD Biosciences, Catalog No. 612803), anti-CD8a (Clone 53-6.7, BUV496, BD Biosciences, Catalog No. 750024), anti-CD4 (Clone GK1.5, APC/Fire™ 810, BioLegend, Catalog No. 100480), anti-Foxp3 (Clone FJK-16s, eFluor450, Invitrogen, Catalog No. 48-5773-82), anti-CD25 (Clone PC61.5, Super Bright 600, Invitrogen, Catalog No. 63-0251-82), anti-Neuropilin-1 (Clone 3DS304M, APC, Invitrogen, Catalog No. 17-3041-82), anti-CD44 (Clone IM7, BUV611, BD Biosciences, Catalog No. 741471), anti-CD62L (Clone MEL-14, Brilliant Violet 421, BioLegend, Catalog No. 104436), anti-PD1 (Clone J43, APC-eflour780, Invitrogen, Catalog No. 47-9985-82), anti-Tim3 (Clone RMT3-23, Brilliant Violet 711, BioLegend, Catalog No. 119727), anti-Klrg1 (Clone 2F1, Pacific Orange, Invitrogen, Catalog No. 79-5893-82), anti-CD27 (Clone LG.3A10, BUV563, BD Biosciences, Catalog No. 741275), anti-CD38 (Clone 90/CD38, Brilliant Violet 750, BD Biosciences, Catalog No. 747103), anti-ICOS (Clone 7E.17G9, Super Bright 436, Invitrogen, Catalog No. 62-9942-82), anti-CD69 (Clone H1.2F3, PE/Cyanine5, BioLegend, Catalog No. 104510), anti-OX-40 (Clone OX-86, Brilliant Violet 650, BD Biosciences, Catalog No. 740545), anti-GITR (Clone MIH44, BUV615, BD Biosciences, Catalog No. 751316), anti-CTLA4 (Clone UC10-4B9, PE/Dazzle™ 594, BioLegend, Catalog No. 106318), anti-Ki67 (Clone B56, BUV395, BD Biosciences, Catalog No. 564071), anti-Tcf1 (Clone C63D9, PE/Cyanine7, Cell Signaling Technology, Catalog No. 90511S), anti-Helios (Clone 22F6, FITC, BioLegend, Catalog No. 137214), anti-Bcl-2 (Clone BCL/10C4, Alexa Fluor 647, BioLegend, Catalog No. 633510), anti-

Granzyme B (Clone QA16A02, Alexa Fluor 700, BioLegend, Catalog No. 372222), anti-T-bet (Clone O4-46, Brilliant Violet 786, BD Biosciences, Catalog No. 564141); ***Immune phenotyping panel:*** Anti-CD45 (Clone 30-F11, Brilliant Violet 510, BioLegend, Catalog No. 103138), anti-CD3 (Clone 17A2, BUV737, BD Biosciences, Catalog No. 612803), anti-CD8a (Clone 53-6.7, BUV496, BD Biosciences, Catalog No. 750024), anti-CD4 (Clone GK1.5, APC/Fire™ 810, BioLegend, Catalog No. 100480), anti-Foxp3 (Clone FJK-16s, eFluor450, Invitrogen, Catalog No. 48-5773-82), anti-CD25 (Clone PC61.5, Super Bright 600, Invitrogen, Catalog No. 63-0251-82), anti-CD11b (Clone M1/70, Alexa Fluor 532, Invitrogen, Catalog No. 58-0112-82), anti-F4-80 (Clone T45-2342, BUV395, BD Horizon, Catalog No. 565614), anti-CD11c (Clone N418, Brilliant Violet 750, BioLegend, Catalog No. 117357), anti-MHC-II (Clone M1/42, BUV615, BD Biosciences, Catalog No. 751123), anti-NK-1.1 (Clone PK136, Brilliant Violet 570, BioLegend, Catalog No. 108733), anti-Ly-6C (Clone HK1.4, Brilliant Violet 605, BioLegend, Catalog No. 128036), anti-Ly-6G (Clone 1A8-Ly6g, Super Bright 436, Invitrogen, Catalog No. 62-9668-82), anti-CD103 (Clone 2E7, Brilliant Violet 711, BioLegend, Catalog No. 121435), anti-PD1 (Clone J43, FITC, Invitrogen, Catalog No. 11-9985-82), anti-PD-L1 (Clone 10F.9G2, Brilliant Violet 421, BioLegend, Catalog No. 124315), anti-CD206 (Clone MR6F3, APC-eflour780, Invitrogen, Catalog No. 47-2061-82), anti-CD38 (Clone 90/CD38, PE/Cyanine7, BioLegend, Catalog No. 102718), anti-Arginase 1 (Clone A1exF5, Alexa Fluor 700, Invitrogen, Catalog No. 56-3697-82), anti-CD64 (Clone X54-5/7.1, APC, BioLegend, Catalog No. 139306), XCR1 (Clone ZET, PerCP/Cyanine5.5, BioLegend, Catalog No. 148208), anti-CD172 (Clone P84, PE/Dazzle™ 594, BioLegend, Catalog No. 144016), anti-CD19 (Clone 6D5, Spark NIR™ 685, BioLegend, Catalog No. 115568), anti-CD24 (Clone M1/69, BV480, BD Biosciences, Catalog No. 746709); ***T cell exhaustion panel:*** Anti-CD45 (Clone 30-F11, Brilliant Violet 510, BioLegend, Catalog No. 103138), anti-CD3 (Clone 17A2, BUV737, BD Biosciences, Catalog No. 612803), anti-CD8a (Clone 53-6.7, BUV496, BD Biosciences, Catalog No. 750024), anti-CD4 (Clone GK1.5, APC/Fire™ 810, BioLegend, Catalog No. 100480), anti-Foxp3 (Clone FJK-16s, eFluor450,

Invitrogen, Catalog No. 48-5773-82), anti-CD25 (Clone PC61.5, Super Bright 600, Invitrogen, Catalog No. 63-0251-82), anti-TOX (Clone REA473, PE, Miltenyi Biotec, Catalog No. 130-120-716), anti-CD44 (Clone IM7, BUV611, Invitrogen, Catalog No. 741471), anti-CD62L (Clone MEL-14, Brilliant Violet 421, BioLegend, Catalog No. 104436), anti-Slamf6 (Clone 13G3-19D, APC, Invitrogen, Catalog No. 17-1508-82), anti-PD1 (Clone J43, APC-eflour780, Invitrogen, Catalog No. 47-9985-82), anti-Tim3 (Clone RMT3-23, Brilliant Violet 711, BioLegend, Catalog No. 119727), anti-Lag3 (Clone C9B7W, BUV 805, BD Biosciences, Catalog No. 748540), anti-Klrg1 (Clone 2F1, Pacific Orange, Invitrogen, Catalog No. 79-5893-82), anti-CD27 (Clone LG.3A10, BUV563, BD Biosciences, Catalog No. 741275), anti-CD38 (Clone 90/CD38, Brilliant Violet 750, BD Biosciences, Catalog No. 747103), anti-ICOS (Clone 7E.17G9, Super Bright 436, Invitrogen, Catalog No. 62-9942-82), anti-CD69 (Clone H1.2F3, PE/Cyanine5, BioLegend, Catalog No. 104510), anti-TIGIT (Clone 1G9, Brilliant Violet 650, BD Optibuild, Catalog No. 744213), anti-GITR (Clone MIH44, BUV615, BD Biosciences, Catalog No. 751316), anti-CTLA4 (Clone UC10-4B9, PE/Dazzle™ 594, BioLegend, Catalog No. 106318), anti-CD95 (Clone Jo2, BV480, BD Biosciences, Catalog No. 746755), anti-Ki67 (Clone B56, BUV395, BD Biosciences, Catalog No. 564071), anti-Tcf1 (Clone C63D9, PE/Cyanine7, Cell Signaling Technology, Catalog No. 90511S), anti-Bcl-2 (Clone BCL/10C4, Alexa Fluor 647, BioLegend, Catalog No. 633510), anti-Granzyme B (Clone QA16A02, Alexa Fluor 700, BioLegend, Catalog No. 372222), anti-T-bet (Clone O4-46, Brilliant Violet 786, BD Biosciences, Catalog No. 564141); ***Other panels:*** Anti-TNFα (Clone MP6-XT22, Percp-eflour 710, Invitrogen, Catalog No. 46-7321-82), anti-IFNγ (Clone XMG1.2, Brilliant Violet 786, BD Biosciences, Catalog No. 563773), anti-CD18 (Clone M18/2, PE, BioLegend, Catalog No. 101407), anti-CD11a (Clone M17/4, APC, BioLegend, Catalog No. 101119), anti-CD103 (Clone 2E7, Brilliant Violet 711, BioLegend, Catalog No. 121435), anti-integrin β7 (Clone FIB504, FITC, BioLegend, Catalog No. 321213), anti-CD61 (Clone 2C9.G2/HMβ3-1, APC, BioLegend, Catalog No. 104315), anti-CD51 (Clone RMV-7, PE, BioLegend, Catalog No. 104105), anti-CD29 (Clone TS2/16, Percp-eflour 710, Invitrogen,

Catalog No. 46-0299-42), anti-CD49d (Clone R1-2, FITC, BioLegend, Catalog No. 103605), anti-gp96 (Clone 9G10, PE, Enzo Life Sciences, Catalog No. ADI-SPA-850PE-F), anti-CCR7 (Clone 4B12, PE, BD Pharmingen, Catalog No. 560682), anti-CCR4 (Clone 2G12, PE, BioLegend, Catalog No. 131203), anti-CCR9 (Clone CW-1.2, PE, BioLegend, Catalog No. 128709), anti-CXCR3 (Clone CXCR3-173, APC, BioLegend, Catalog No. 126511), anti-CCR6 (Clone 140706, Brilliant Violet 711, BD OptiBuild, Catalog No. 740646), anti-CCR2 (Clone SA203G11, Brilliant Violet 421, BioLegend, Catalog No. 150605), anti-CX3CR1 (Clone SA011F11, APC/Cyanine7, BioLegend, Catalog No. 149047), anti-CXCR5 (Clone L138D7, APC/Cyanine7, BioLegend, Catalog No. 145525), anti-pSTAT5 (pY647, clone 47, PE, BD Phosflow, Catalog No. 562077).

For cytokine production experiment, cells were stimulated with 5 μg/ml plate-bound anti-CD3 (Clone 17A2, BioLegend, Catalog No. 100202) and 2.5 μg/ml soluble anti-CD28 (Clone 37.51, BioLegend, Catalog No. 102121) in the presence of brefeldin A (BioLegend) in a 48-well plate at 37°C for 5 hours. For intracellular gp96 staining, after the live/dead and surface markers staining, cells were fixed with 4% paraformaldehyde (PFA, Thermo Fisher Scientific) for 10 minutes at RT and permeabilized by 100% methanol (Sigma) at - 20°C for 10 minutes, followed by overnight staining of intracellular gp96 and Foxp3 at 4°C. For *in vitro* STAT5 phosphorylation assay, cells were stimulated with or without recombinant human IL-2 (rhIL-2, R&D Systems) for indicated time and subjected immediately to fixation and permeabilization with 4% PFA and 90% methanol, respectively. Both surface and intracellular staining were subsequently performed at RT for 1 hour. For *ex vivo* STAT5 phosphorylation assay, tissues including SPLs, pLNs and tumors were directly disrupted into BD Cytofix/Cytoperm (BD Biosciences) and incubated at RT for 30 minutes. Single-cell suspensions were harvested using 70-mm strainers (Thermo Fisher Scientific) and permeabilized with 90% methanol at 4°C for 30 min. After that, surface and intracellular staining were performed at RT for 1 hour. For the EdU cell proliferation

assay, both WT and KO mice received daily intravenous administration of 200 μg EdU (5-ethynyl-2´-deoxyuridine) from day -5 (D-5) to day -1 (D-1) during the 10-day tamoxifen treatment. On day 0 (D0), splenocytes from the mice were harvested, and the frequency of EdU^+ proliferating Tregs were assessed using Click-iT™ Plus EdU Alexa Fluor™ 647 kit (Invitrogen).

In-vitro iTreg differentiation and Crispr/Cas9 electroporation:

Naïve $CD4^+$ T cells were freshly isolated from SPLs of JAX-WT mice using Mouse Naive $CD4^+$ T Cell Isolation Kit (Miltenyi Biotec) according to its protocol. Afterwards, cells were stimulated under Treg-skew conditions with 5 μg/mL plate-bound anti-CD3 antibody (Clone 17A2, BioLegend, Catalog No. 100202), 2.5 μg/mL soluble anti-CD28 antibody (Clone 37.51, BioLegend, Catalog No. 102121), 5 ng/mL TGF-β1 (R&D Systems), and 2000 IU/mL rhIL-2 (R&D Systems) for 5 days. On day 2 (D2) during iTreg differentiation, crispr/cas9 electroporation was performed to delete indicated integrins in iTregs using Lonza 4D-NucleofectorTM System (83, 84). Briefly, Cas9/RNP complexes (Integrated DNA Technologies, IDT) were prepared at 3:1 ratio of sgRNA (1.5 μl per target) to Alt-R® S.p. HiFi Cas9 Nuclease V3 (2 μl per target) and incubated at RT for 10 minutes. For efficient deletion of individual targets, two specific pre-validated gRNAs were used for each gene (please find the detailed gRNAs sequences in **Extended Data Table 8**). In parallel, iTregs were suspended at $2x10^6$ cells in 20ul RT P4 nucleofection buffer (Lonza) and 4uM of Alt-R® Cas9 Electroporation Enhancer (IDT) was added per sample. Cells/enhancer mixture was incubated with 7 μl Cas9/RNP complexes at RT for 2 min in a round bottom 96-well plate and then transferred to Nucleofection cuvette strips (4D-Nucleofector X kit S; Lonza). Cells were nucleofected using program CM137 on the 4D-Nucleofector system (Lonza). After electroporation, cells were allowed to recover for 2 hr in the 96-well plate at 37°C incubator, then cultured continually under Treg-skew conditions in a pre-warmed 24-well plate for 3 days. The KO efficiency was determined on D5 by flow cytometry.

In vitro Treg suppression assay:

Murine spleen-derived non-regulatory $CD4^+$ Teffs and $CD8^+$ T cells were used as responder T cells. $CD4^+$ Teffs were enriched using EasySep™ Mouse $CD4^+$ T Cell Isolation Kit (Stem Cell Technologies), followed by depletion of Tregs using EasySep™ Mouse $CD4^+CD25^+$ Regulatory T Cell Isolation Kit II (Stem Cell Technologies). $CD8^+$ T cells were isolated using EasySep™ Mouse $CD8^+$ T Cell Isolation Kit (Stem Cell Technologies). Both $CD4^+$ Teffs and $CD8^+$ T cells were then labeled with CellTrace Violet (CTV, Invitrogen). Splenic Tregs (suppressors) were purified by FACS based on fluorescence tdTomato protein from WT-tdTomato or KO-tdTomato mice. Before FACS sorting, total $CD4^+$ T cells were pre-enriched by EasySep™ Mouse $CD4^+$ T Cell Isolation Kit (Stem Cell Technologies). Co-cultures were set up with either 5×10^4 $CD4^+$ Teffs or 1×10^4 $CD8^+$ T cells along with WT or KO Tregs at indicated ratios, in the presence of 5 μg/mL anti-CD3 antibody (Clone 17A2, BioLegend, Catalog No. 100202) and 2.5 μg/mL anti-CD28 antibody (Clone 37.51, BioLegend, Catalog No. 102121) in a 96-well plate. Cell proliferation of responder $CD4^+$ Teffs or $CD8^+$ T cells was determined by flow cytometry based on their dilutions of CTV fluorescence intensity after stimulation for the designated duration.

Adoptive transfer model:

For adoptively transferring integrin-deficient iTregs experiment, nucleofected iTregs ($2x10^6$) containing specific-targeting sgRNAs or non-targeting control sgRNAs along with CAS9 were intravenously injected (i.v.) vial tail into a *Rag2*$^{-/-}$ recipient mouse on D3 post MC38 implantation. Seven days later (D10), mice were sacrificed by CO2 asphyxiation, and tumors and SPLs were harvested for ex vivo analysis of Tregs frequencies.

For adoptively transferring WT and gp96-null Tregs experiment, splenic $CD4^+$ T cells were enriched by EasySep™ Mouse $CD4^+$ T Cell Isolation Kit (Stem Cell Technologies) and

thereafter tdTomato$^+$ Tregs were isolated by FACS from SPLs of TdTomato-WT and TdTomato-KO mice having received 10-day tamoxifen treatment. Isolated Tregs were subsequently activated and expanded *in vitro* using mouse Treg Expansion Kit (Miltenyi Biotec) together with 2000 U/mL rhIL-2 (R&D Systems) for 3 days. Pre-activated tdTomato$^+$ WT or gp96 KO Tregs (2x10^6 per mouse) were then adoptively transferred via tail i.v. into *Rag2*$^{-/-}$ recipient mice on D3 post MC38 inoculation. Seven days later (D10), Treg accumulation in the SPL and the tumor was determined by flow cytometry.

For co-transferring Tregs and CD8$^+$ T cells experiment, tdTomato$^+$ WT or gp96 KO Tregs were isolated and pre-activated as mentioned above and adoptively transferred into MC38-bearing *Tcrbd*$^{-/-}$ recipient mice 2 days (D2) after tumor implantation. In parallel, CD8$^+$ T cells were isolated by FACS from draining inguinal lymph nodes (dLNs) of day-12 MC38-bearing JAX-WT mice and stimulated with 5 μg/mL plate-bound anti-CD3 antibody (Clone 17A2, BioLegend, Catalog No. 100202), 2.5 μg/mL soluble anti-CD28 antibody (Clone 37.51, BioLegend, Catalog No. 102121), and 100 IU/mL rhIL-2 (R&D Systems) for 3 days. Donor CD8$^+$ T cells were intravenously transferred into MC38-bearing *Tcrbd*$^{-/-}$ recipient mice on D4 post tumor implantation. The tumor growth in *Tcrbd*$^{-/-}$ recipient mice were monitored at indicated time points.

RNA sequencing:

1×10^6 of splenic GFP$^+$ Tregs were purified from WT (n=4) and KO mice (n=4) via FACS isolation. RNA was extracted using RNeasy Micro Kit (Qiagen) following its standard protocol and RNA degradation and contamination was monitored on 1% agarose gels. For RNA sequencing, libraries were prepared using NEBNext® Ultra TM RNA Library Prep Kit for Illumina® (NEB, USA) following manufacturer's recommendations and index codes were added to attribute sequences to each sample. The clustering of the index-coded samples was

performed on a cBot Cluster Generation System using PE Cluster Kit cBot-HS (Illumina) according to the manufacturer's instructions. After cluster generation, the library preparations were sequenced on an Illumina platform and paired-end reads were generated. All samples were prepared at the same time and sequenced on the same lane. Original image data file from high-throughput sequencing platforms (like Illumina) is transformed to sequenced reads (called Raw Data or Raw Reads) by CASAVA base recognition (Base Calling). Raw data in FASTQ format were processed through fastp. In this step, clean data (clean reads) were obtained by removing reads containing adapter and poly-N sequences and reads with low quality from raw data. All the downstream analyses were based on the clean data with high quality. Reference mouse genome and gene model annotation files were downloaded from genome website browser (NCBI/UCSC/Ensembl). Paired-end clean reads were mapped against the reference genome using the Spliced Transcripts Alignment to a Reference (STAR) software. FeatureCounts was used to count the read numbers mapped of each gene. And then RPKM (Reads Per Kilobase of exon model per Million mapped reads) of each gene was calculated based on the length of the gene and reads count mapped to this gene. Differential expression analysis between two groups (four biological replicates per group) was performed using DESeq2 R package (version 3.1.0). The resulting *P* values were adjusted using the Benjamini and Hochberg's approach for controlling the False Discovery Rate (FDR). Genes with an adjusted *P* value < 0.05 found by DESeq2 were assigned as differentially expressed. A common way for searching shared functions among genes is to incorporate the biological knowledge provided by biological ontologies. Gene Ontology (GO) enrichment analysis of differentially expressed genes was implemented by the clusterProfiler R package. GO terms with corrected *P* value < 0.05 were considered significantly enriched by differential expressed genes. Gene Set Enrichment Analysis (GSEA) was performed using the GSEA desktop (v4.0.3) and all parameters were followed by default settings.

RNA integrity and quantitation were assessed using the RNA Nano 6000 Assay Kit of the Bioanalyzer 2100 system (Agilent Technologies, CA, USA). For RNA sequencing, libraries were prepared using NEBNext® Ultra TM RNA Library Prep Kit for Illumina® (NEB, USA; Catalogue#: E7530) following manufacturer's recommendations and index codes were added to attribute sequences to each sample. The clustering of the index-coded samples was performed on a cBot Cluster Generation System using PE Cluster Kit cBot-HS (Illumina) according to the manufacturer's instructions. After cluster generation, the library preparations were sequenced on an Illumina platform and paired-end reads were generated. All samples were prepared at the same time and sequenced on the same lane. Original image data file from high-throughput sequencing platforms (like Illumina) is transformed to sequenced reads (called Raw Data or Raw Reads) by CASAVA base recognition (Base Calling). Raw data in FASTQ format were processed through fastp. In this step, clean data (clean reads) were obtained by removing reads containing adapter and poly-N sequences and reads with low quality from raw data. All the downstream analyses were based on the clean data with high quality. Reference mouse genome and gene model annotation files were downloaded from genome website browser (NCBI/UCSC/Ensembl). Paired-end clean reads were mapped against the reference genome using the Spliced Transcripts Alignment to a Reference (STAR) software. FeatureCounts was used to count the read numbers mapped of each gene. And then RPKM (Reads Per Kilobase of exon model per Million mapped reads) of each gene was calculated based on the length of the gene and reads count mapped to this gene. Differential expression analysis between two groups (four biological replicates per group) was performed using DESeq2 R package (version 3.1.0). The resulting *P* values were adjusted using the Benjamini and Hochberg's approach for controlling the False Discovery Rate (FDR). Genes with an adjusted P value < 0.05 found by DESeq2 were assigned as differentially expressed. A common way for searching shared functions among genes is to incorporate the biological knowledge provided by biological ontologies. Gene Ontology (GO) enrichment analysis of differentially expressed genes was

implemented by the clusterProfiler R package. GO terms with corrected *P* value < 0.05 were considered significantly enriched by differential expressed genes. Gene Set Enrichment Analysis (GSEA) was performed using the GSEA desktop (v4.0.3) and all parameters were followed by default settings.

Gene correlation, immune deconvolution and differential integrin alpha expression analysis:

Correlation analysis of mRNA levels between FOXP3 and T cell exhaustion signatures including HAVCR2 (TIM3), PDCD1 (PD1), TIGIT, LAG3, CTLA4, CXCL13, TOX, and CD8A across diverse TCGA (The Cancer Genome Atlas) treatment-naïve cancers was performed using the online Gene Expression Profiling Interactive Analysis 2 (GEPIA2) webserver (http://gepia2.cancer-pku.cn/#index) following the instructions (85). The T cell exhaustion signature score was visualized as mean value of the log2 (Transcripts per million, TPM, +1) of each gene and spearman correlation was subsequently selected for calculating the correlation coefficient between FOXP3/signature score.

For comparison of alpha integrin mRNA expression in tumor infiltrating versus peripheral Tregs, we reanalyzed the normalized TPM values for integrin alpha members (*ITGAD* (CD11D), *ITGAE* (CD103), *ITGAM* (CD11B), *ITGAL* (CD11A), *ITGAV* (CD51), *ITGAX* (CD11C), *ITGA1*, *ITGA2* (CD49B), *ITGA2B* (CD41), *ITGA3* (CD49C), *ITGA4* (CD49D), *ITGA5* (CD49E), *ITGA6* (CD49F), *ITGA7, ITGA8, ITGA9, ITGA10* and *ITGA11*) from the previously published dataset under accession GSE89225 (27). For immune deconvolution analysis, normalized mRNA expression TPM values accessed via the Firehose Broad GDAC database for ITGAL were used to stratify patients from the TCGA Breast Invasive Carcinoma, Skin Cutaneous Melanoma, and Colorectal Adenocarcinoma cohorts into ITGAL high patients (upper quartile of normalized ITGAL mRNA expression) and ITGAL low patients (lower quartile of normalized ITGAL mRNA expression);

ITGAL Low and ITGAL High patient normalized TMP mRNA values were then processed via the TIMER2.0 interface to generate immune infiltrate estimation values. For survival analysis, patients from the TCGA cohorts Colorectal Adenocarcinoma (TCGA, PanCancer Atlas), Uveal Melanoma (TCGA, PanCancer Atlas), Brain Lower Grade Glioma (TCGA, PanCancer Atlas), and Kidney Renal Clear cell Carcinoma (TCGA, PanCancer Atlas) were assigned as ITGAL high for samples with mRNA expression z-scores >1.75 relative to all samples (log RNA Seq V2 RSEM); all other samples were designated as ITGAL low. Overall survival values and mRNA z-scores were accessed via the cBioportal for cancer genomics.

Extended Figures

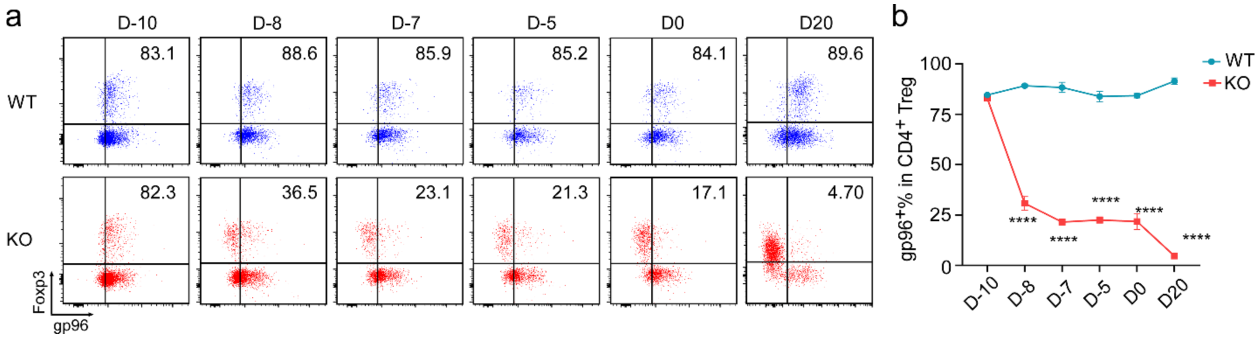

Figure S1. Frequencies of gp96 expressing Treg cells in the spleens of WT and KO mice. a-b. Representative flow cytometry plots (**a**) and summary graphs (**b**) illustrate the frequency of splenic gp96^{+} Tregs at designated time points following tamoxifen injection (day -10 to 0) in WT and KO mice (n=3/group at each time point). Numbers in the flow plots indicate the percentages of gp96^{+} subsets within Foxp3^{+} Tregs. Results are representative of more than three independent experiments. Data were shown as means ± SEM. ****p<0.0001 (KO versus WT). Two-tailed Student's t test was used for comparisons of different experimental groups (S1b).

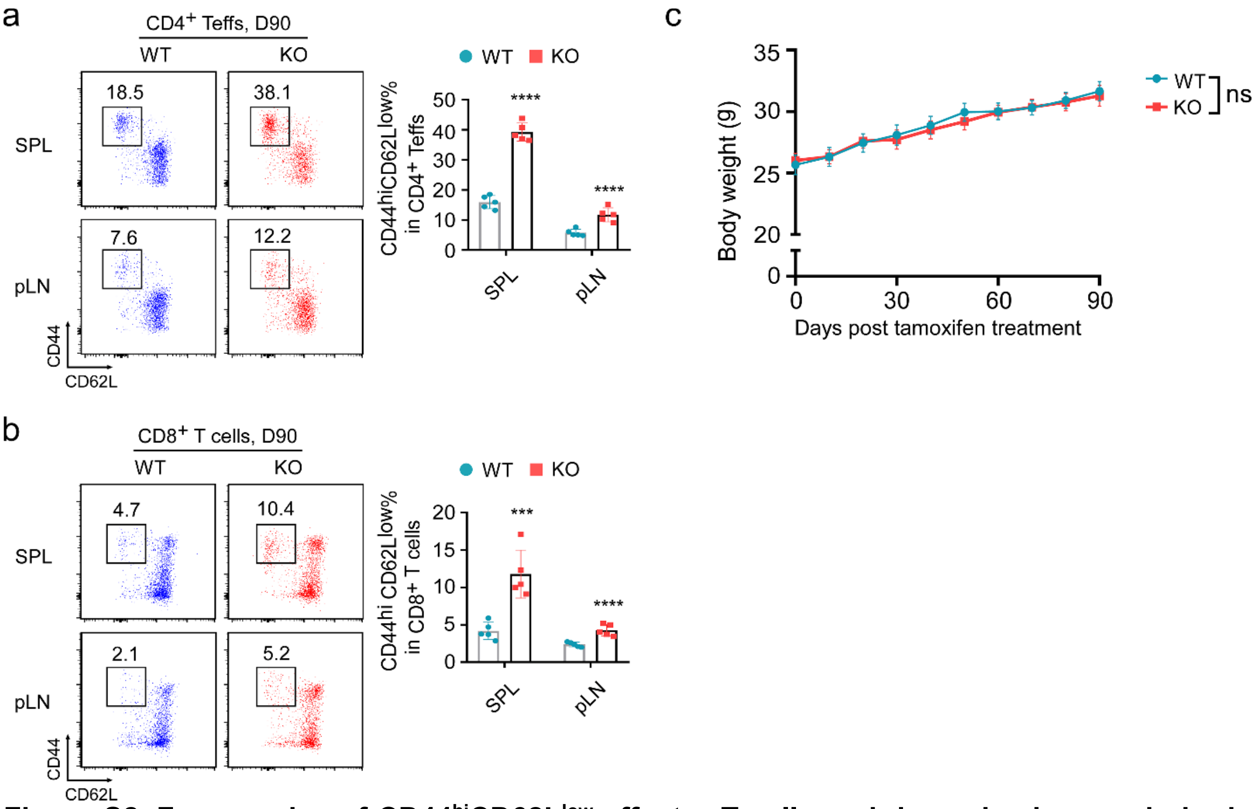

Figure S2. Frequencies of CD44^{hi}CD62L^{low} effector T cells and dynamic changes in body weight upon Treg-specific gp96 deletion in mice. a-b, Representative flow cytometry plots (**left**) and summary graphs (**right**) depict the percentages (with numbers indicated) of CD44^{hi}CD62L^{low} subsets within CD4^{+} Teffs (**a**) and CD8^{+} T cells (**b**) from the SPL and pLN of WT and KO mice (n=5/group) on day 90 (D90) after a 10-day intraperitoneal (i.p.) injection of tamoxifen (day -10 to 0). **c**. Summary graph depicts the dynamic changes in body weight up to 90 days following a 10-day tamoxifen treatment in both WT and KO mice (n=11/group). Results are representative of more than three independent experiments. Data were shown as means ± SEM. ns: not significant, ***p<0.001, ****p<0.0001 (KO versus WT). Two-tailed Student's t test was used for comparisons of different experimental groups (S2a-b). Body weight curves were analyzed by repeated measurements two-way ANOVA (S2c).

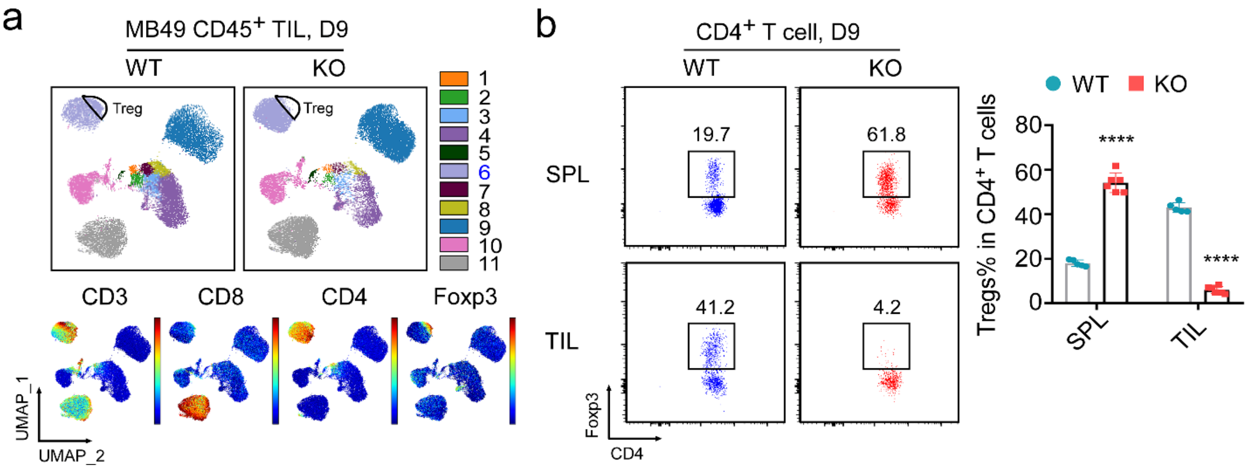

Figure S3. Distinct immune phenotype of CD45⁺ TILs by Treg-specific gp96 deletion in MB49-bearing mice. a, Spectral flow cytometry analysis of $CD45^+$ TILs from WT and KO mice (n=5-6/group; pretreated with tamoxifen from day -10 to 0) on day 9 (D9) post MB49 implantation. The distribution of CD3, CD8, CD4 and Foxp3 expression was indicated (**bottom**). Cluster 6 (including $CD3^+CD4^+Foxp3^-$ non-Tregs and $CD3^+CD4^+Foxp3^+$ Tregs) was highlighted in blue on the right side; Tregs (cycled) are positioned in the upper right of cluster 6. **b**, Representative flow cytometry plots (**left**) and summary graph (**right**) show frequencies (with numbers indicated) of $Foxp3^+$ Tregs in gated $CD4^+$ T cells from both SPL and TIL on day 9 (D9) in MB49 tumors of WT and KO mice (same as **a**). Results are representative of more than three independent experiments. Data were shown as means ± SEM. ****p<0.0001 (KO versus WT). Two-tailed Student's t test was used for comparisons of different experimental groups (S3b).

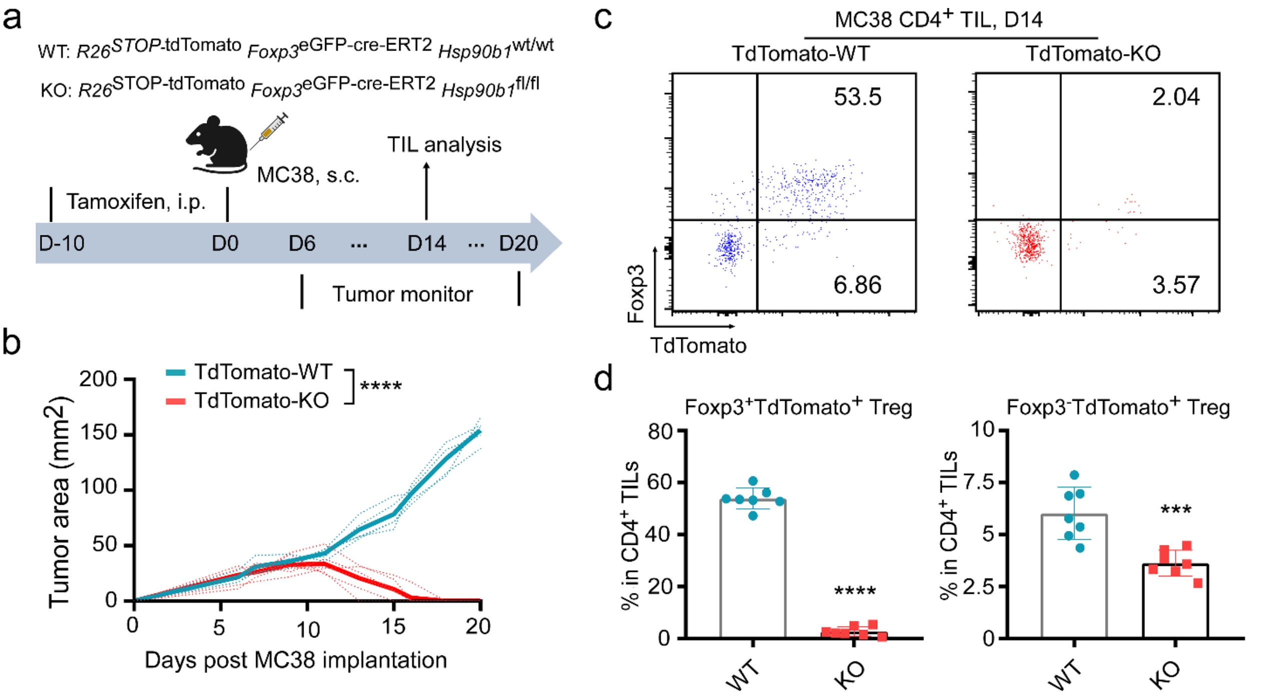

Figure S4. Treg-specific gp96 deletion exhibits superior MC38 tumors control by limiting Tregs infiltration into the TME in TdTomato-KO mice. a, Experimental schema outlines the process of MC38 implantation (2x10^6, s.c.) on day 0 (D0) in TdTomato-WT (*$R26^{STOP\text{-}tdTomato}$$Foxp3^{eGFP\text{-}Cre\text{-}ERT2}$$Hsp90b1^{wt/wt}$*) and TdTomato-KO (*$R26^{STOP\text{-}tdTomato}$$Foxp3^{eGFP\text{-}Cre\text{-}ERT2}$$Hsp90b1^{fl/fl}$*) mice receiving tamoxifen treatment (day -10 to 0). Tumor growth was monitored daily or every two days starting from D6 post MC38 inoculation. On D14, Tumors were collected, and TIL analysis was performed. **b**, Tumor growth curve of MC38 in TdTomato-WT and TdTomato-KO mice (n=5-6/group). Tumor sizes were measured as described above**. c-d**, Representative flow cytometry plots (**c**) and summary graphs (**d**) show the percentages (with numbers indicated) of $Foxp3^+tdTomato^+$ and $Foxp3^-tdTomato^+$ Tregs in gated $CD4^+$ TILs on day 14 of MC38 tumors in TdTomato-WT and TdTomato-KO mice (n=7/group). Results are representative of more than three independent experiments. Data were shown as means ± SEM. ***p<0.001, ****p<0.0001 (KO versus WT). Tumor growth curves were analyzed by repeated measurements two-way ANOVA (S4b). Two-tailed Student's t test was used for comparisons of different experimental groups (S4d).

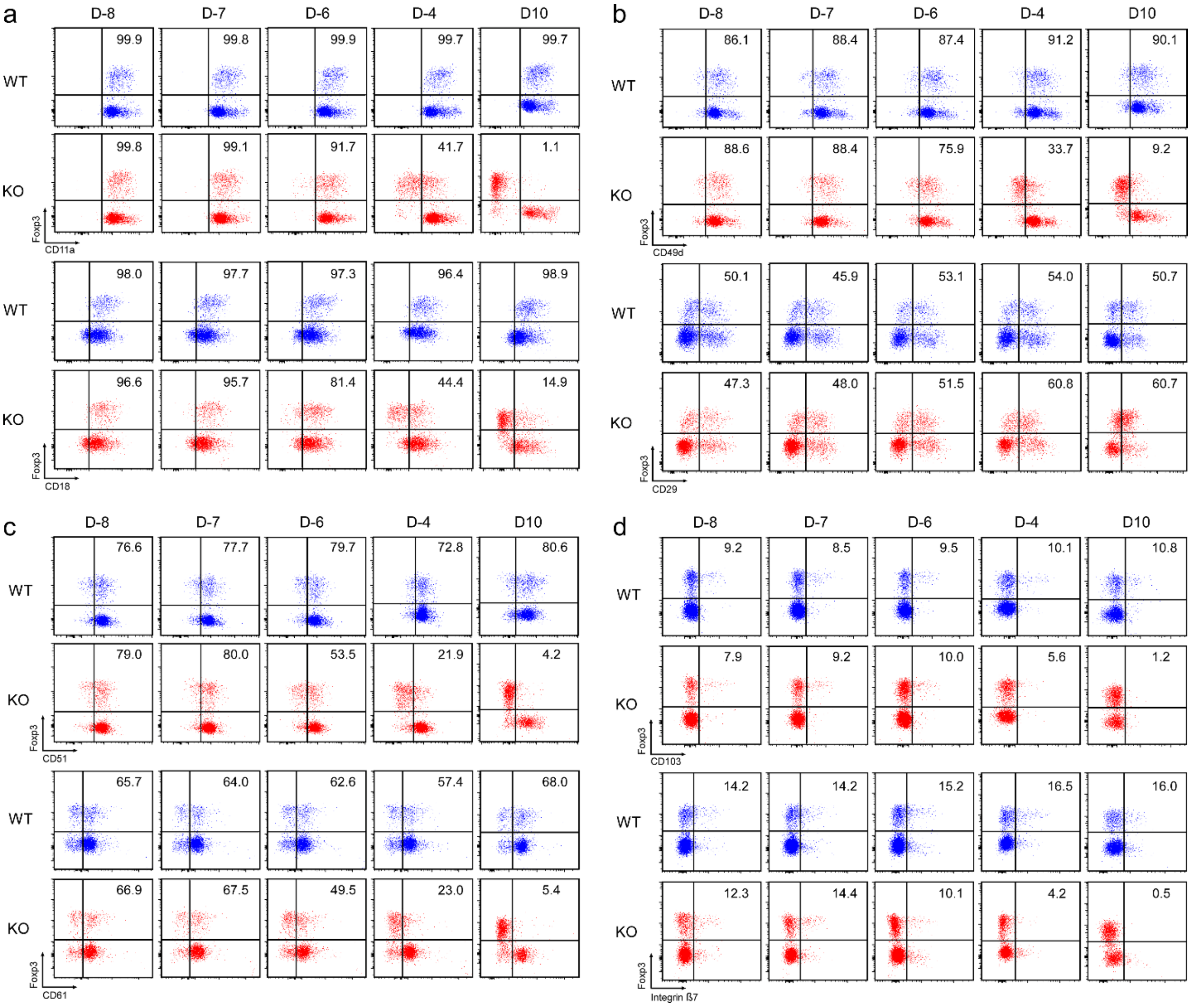

Figure S5. Dynamic changes of surface integrins on Tregs upon gp96 deletion. Representative flow cytometry plots depicts expression of surface CD11a/CD18 (αl/β2; **a**), CD49d/CD29 (α4/β1; **b**), CD51/CD61 (αv/β3; **c**) and CD103/Integrin β7 (αe/β7; **d**) integrins on splenic Tregs at different time points (D-8, D-7, D-6, D-4, and D10) in WT and KO mice (n=3/group at each time point) receiving tamoxifen treatment (days -10 to 0). Numbers shown in the flow plots represent the frequencies of integrin-expressing subsets within Foxp3$^+$ Tregs. Results are representative of three independent experiments.

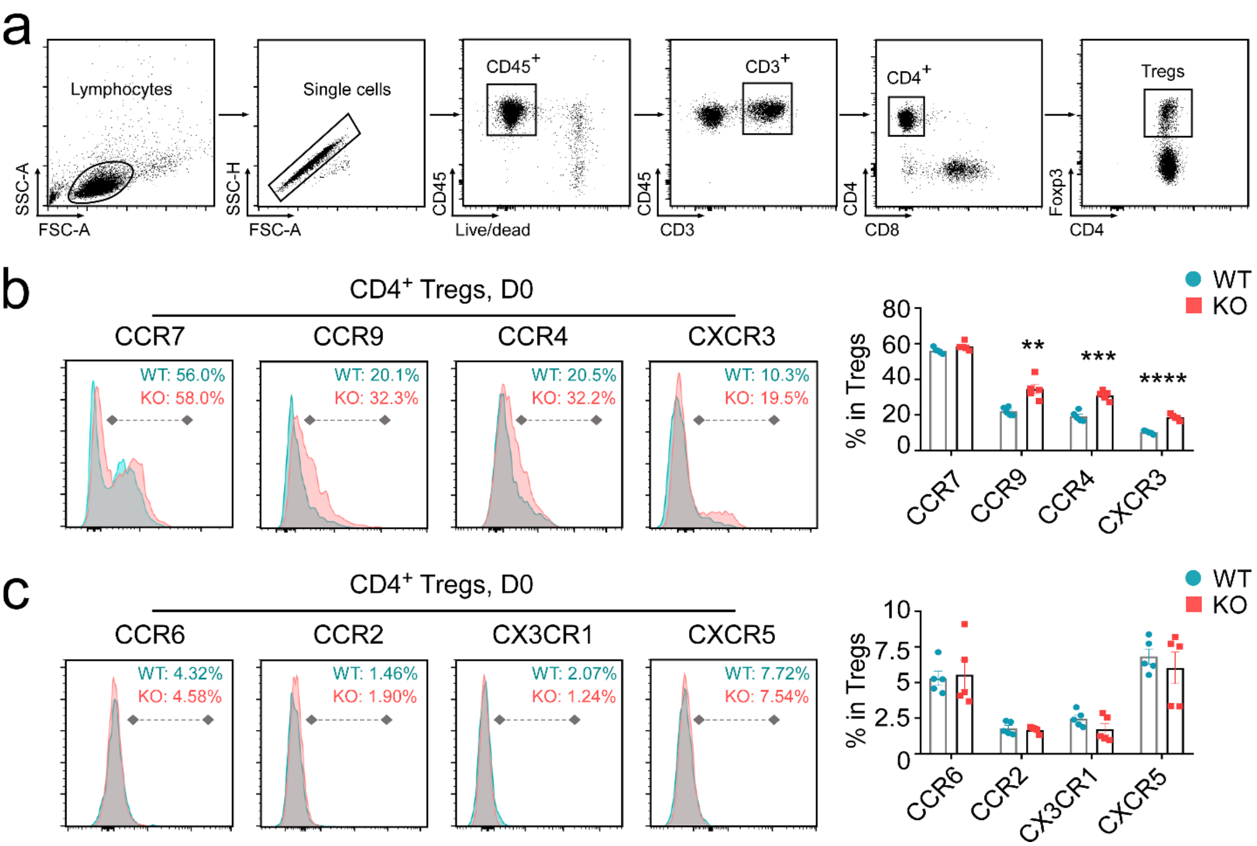

Figure S6. The expression of chemokine receptors in splenic WT and KO Tregs. a, Gating strategy of splenic Tregs by flow cytometry analysis. **b-c,** Representative histograms (**left**) and summary graphs (**right**) depict the expression levels of indicated chemokine receptors in Tregs from spleens of WT and KO mice (n=5/group) receiving a 10-day tamoxifen treatment (days -10 to 0). The frequencies of chemokine receptor-expressing subsets within Foxp3$^+$ Tregs were indicated in the histograms. Results are representative of three independent experiments. Data were shown as means ± SEM. $^{**}p<0.01$, $^{***}p<0.001$, $^{****}p<0.0001$ (KO versus WT). Two-tailed Student's *t* test was used for comparisons of different experimental groups (S5b, c).

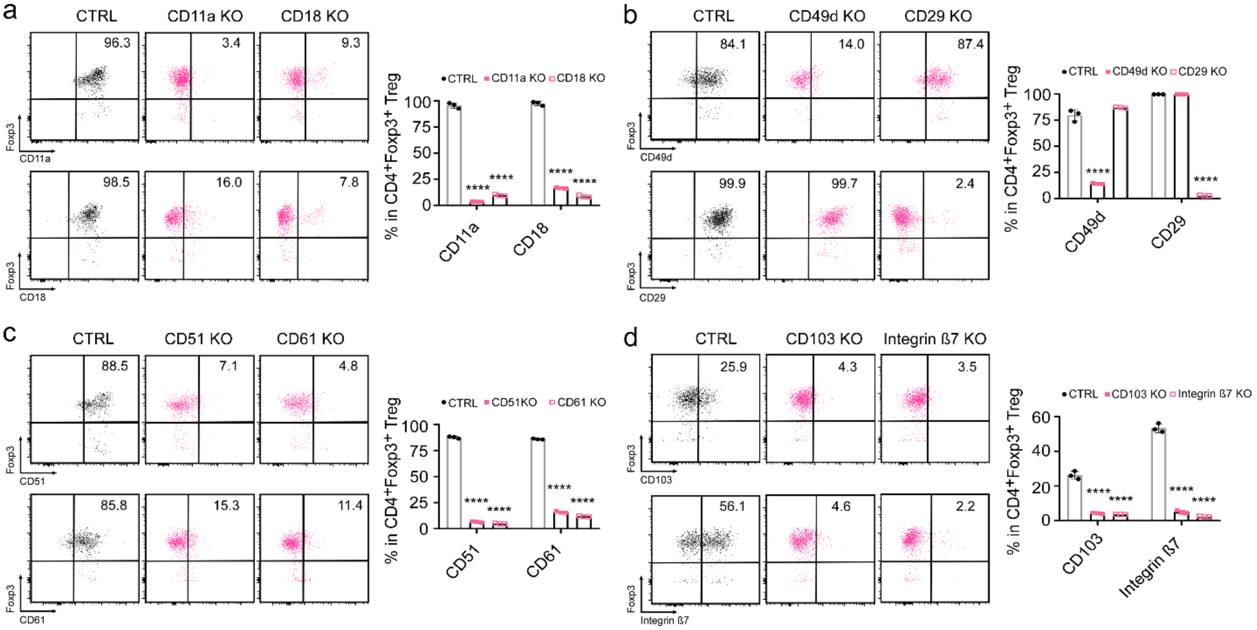

Figure S7. Efficiency of integrins deletion in iTregs using CRISPR/Cas9 electroporation. **a-d**, Representative flow cytometry plots (**left**) and summary graph (**right**) show expression of surface CD11a/CD18 (αl/β2; **a**), CD49d/CD29 (α4/β1; **b**), CD51/CD61 (αv/β3; **c**) and CD103/Integrin β7 (αe/β7; **d**) integrins on iTregs 3 days after CRISPR/Cas9 electroporation (n=3/group). Numbers shown in the flow plots represent the frequencies of integrin-expressing subsets in Foxp3^{+} iTregs; CTRL: non-targeting control iTregs. Results are representative of three independent experiments. Data were shown as means ± SEM. ****p<0.0001 (KO versus WT). One-Way ANOVA was performed with Tukey's multiple comparison correction for comparisons across all groups (S7a-d).

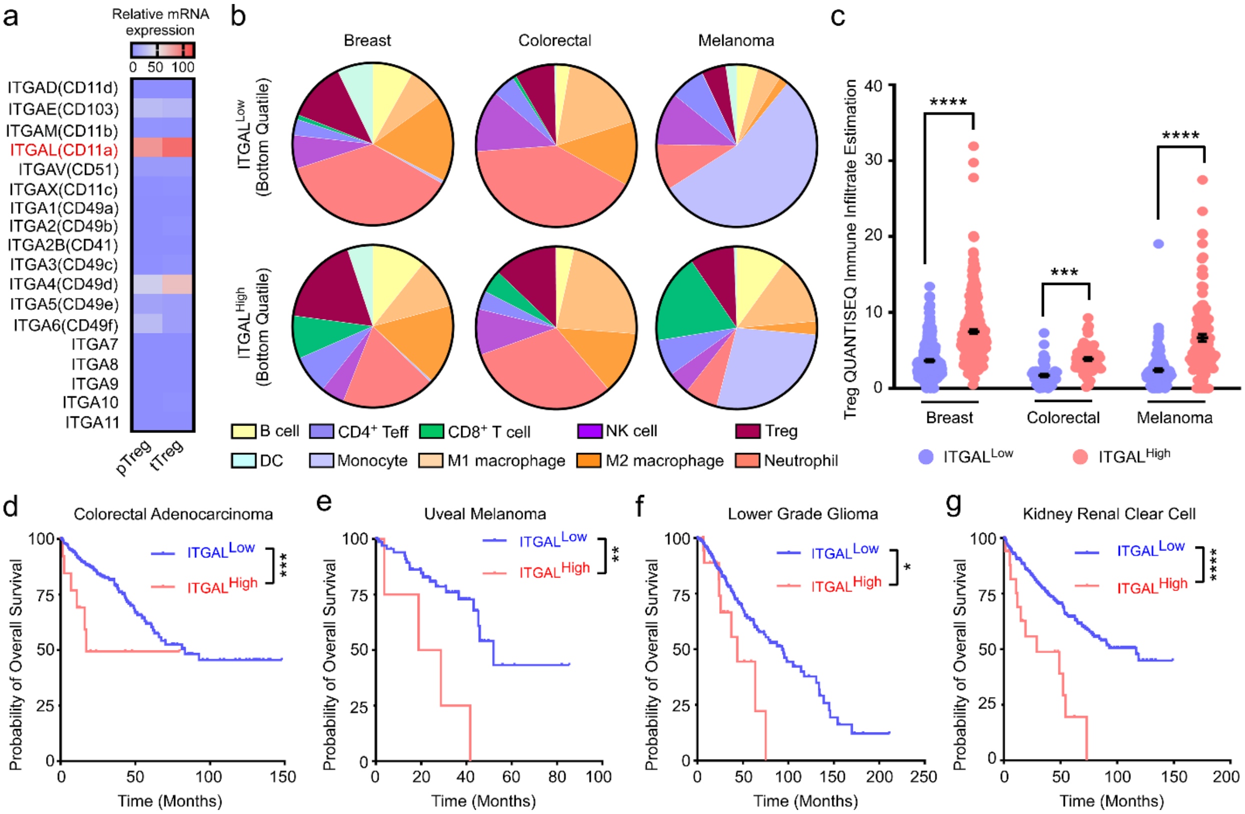

Figure S8. A comprehensive analysis underscoring the critical role of LFA-1 in facilitating Treg infiltration into human cancers and its clinical relevance in overall survival. a, Median mRNA expression of alpha integrin transcripts was compared across peripheral Tregs (pTregs, n = 4) and tumor infiltrating Tregs (tTregs, n = 6) isolated from human breast cancer patients via FACS from the public data set under accession: GSE89225. **b**, Quantiseq immune deconvolution analysis was performed via the Tumor Immune Estimation Resource (TIMER2.0) platform, utilizing normalized transcriptomic expression profiles from the cancer genome atlas (TCGA) breast invasive carcinoma (left), colorectal adenocarcinoma (middle), and skin cutaneous melanoma (right) cohorts with ITGAL/LFA1^{High} patients designated as patients with upper quartile ITGAL mRNA expression and ITGAL/LFA1^{Low} patients designated as patients with lower quartile ITGAL mRNA expression; proportions represent estimated immune subset infiltration relative to all quantitated immune infiltration estimates. **c**, Comparison of estimated tumor infiltrating Tregs from Quantiseq immune deconvolution analysis from TCGA breast invasive carcinoma (left; ITGAL^{High} n = 303, ITGAL^{Low} n = 303), colorectal adenocarcinoma (middle; ITGAL^{High} n = 66, ITGAL^{Low} n= 66), and skin cutaneous melanoma (right; ITGAL^{High} n = 119, ITGAL^{Low} n= 119) cohorts with ITGAL/LFA1^{high} patients designated as patients with upper quartile ITGAL mRNA expression and ITGAL/LFA1^{low} patients designated as patients with lower quartile ITGAL mRNA expression. **d-g**, Comparison of overall survival from ITGAL^{High} and

ITGAL^{Low} patients from the TCGA colorectal adenocarcinoma (**d**, n = 588), uveal melanoma (**e**, n = 80), lower grade glioma (**f**, n = 513) and kidney renal clear cell carcinoma (**g**, n = 510) data sets with ITGAL^{High} status designated as ITGAL mRNA expression z scores > 1.75 and ITGAL^{Low} status designated as ITGAL mRNA expression z scores < 1.75 relative to all samples (log RNA Seq V2 RSEM). Log-rank Mantel-Cox test was performed for comparison of survival curves (S8d-g); for comparison of Quantiseq Treg infiltration, One-Way ANOVA with Sidak's multiple comparison correction was performed (S8c).

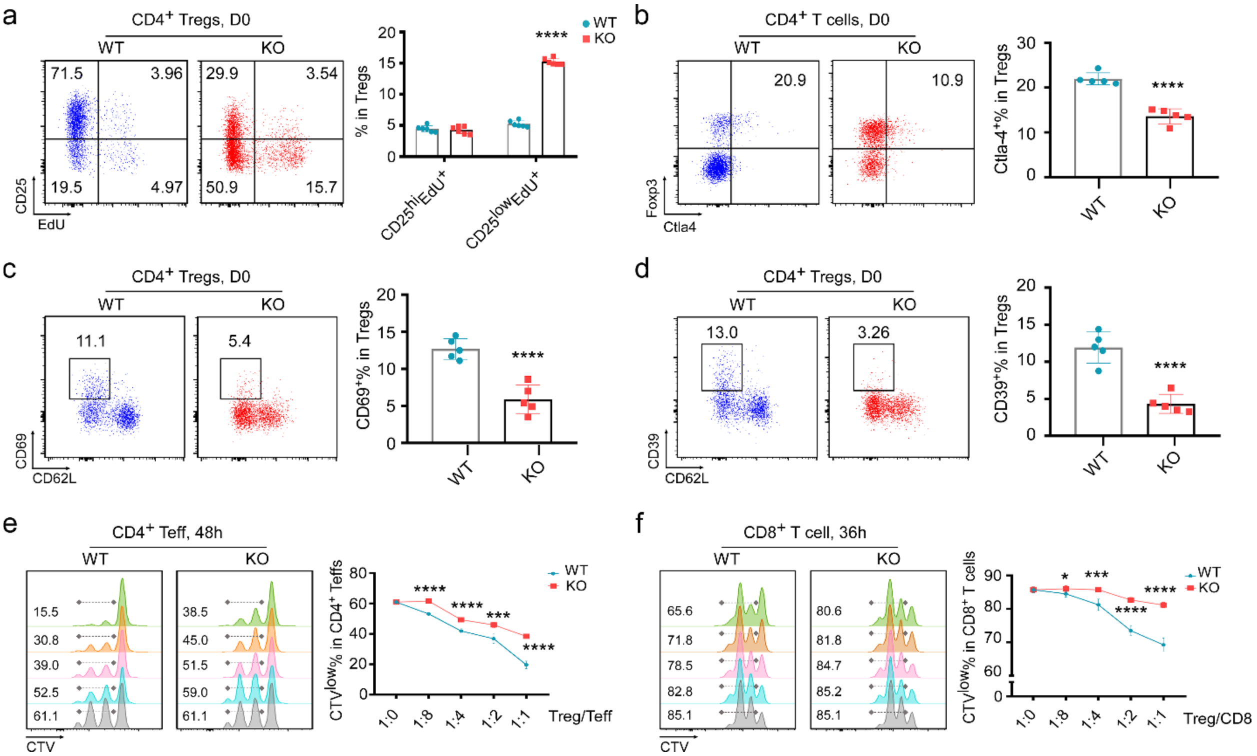

Figure S9. The effects of gp96 deletion on Tregs proliferation and suppressive activity. a-d, Representative flow cytometry plots (**left**) and summary graphs (**right**) illustrate the frequencies (with numbers indicated) of EdU$^+$ Tregs (**a**), Ctla4$^+$ Tregs (**b**), CD69$^+$CD62L^{low} Tregs (**c**), and CD39$^+$CD62L^{low} Tregs (**d**) in the SPLs of WT and KO mice (n=5-6/group) following a 10-day tamoxifen treatment (day -10 to 0) on D0. **e-f**. Representative histograms (**left**) and summary graphs (**right**) show the frequencies of CTV-labeled CD4$^+$ Teffs (**e**) and CD8$^+$ T cells (**f**) when co-cultured with splenic WT or KO Tregs at different ratios for the indicated duration. n=3/group. Results are representative of more than three independent experiments. Data were shown as means ± SEM. ***p<0.001, ****p<0.0001 (KO versus WT). Two-tailed Student's t test was used for comparisons of different experimental groups (S9a-f).

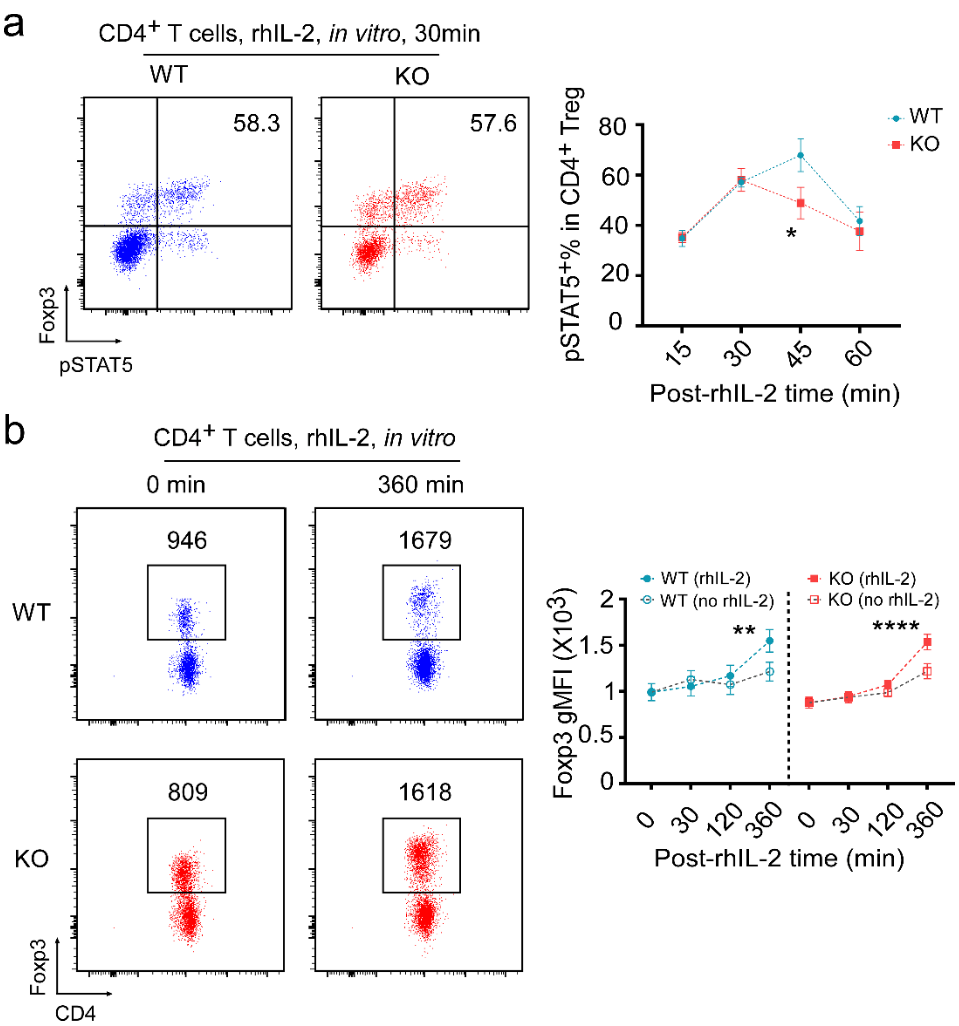

Figure S10. Gp96-null Tregs retain responsiveness to IL-2 stimulation *in vitro*. a, CD4⁺ T cells isolated from SPLs of WT and KO mice treated with a 10-day tamoxifen regimen, were stimulated *in vitro* with recombinant human interleukin-2 (rhIL-2, 500 U/ml) for the indicated time points (15 to 60 min). The phosphorylated STAT-5 (pSTAT-5) level was determined using flow cytometry. Representative flow cytometry plots (**left**) and graph (**right**) show the percentages (with numbers indicated) of pSTAT-5⁺ subset within CD4⁺ Tregs upon rhIL-2 stimulation *in vitro,* comparing the WT and KO groups (n=3/group). **b**, similar to **a,** CD4⁺ T cells were treated *in vitro* with rhIL-2 for up to 360 mins. Representative flow cytometry plots (**left**) and graph (**right**) illustrate Foxp3 geometric mean fluorescent intensity (gMFI) in CD4⁺Foxp3⁺ Tregs following rhIL-2 stimulation for the indicated time course. Results are representative of more than three independent experiments. Data were shown as means ± SEM. **p*<0.05 (KO versus WT in **a**), ***p*<0.01 and *****p*<0.0001 (rhIL-2 stimulation for 360 mins versus 0 min in **b**). Two-tailed Student's *t* test was used for comparisons of different experimental groups (S10a, b).

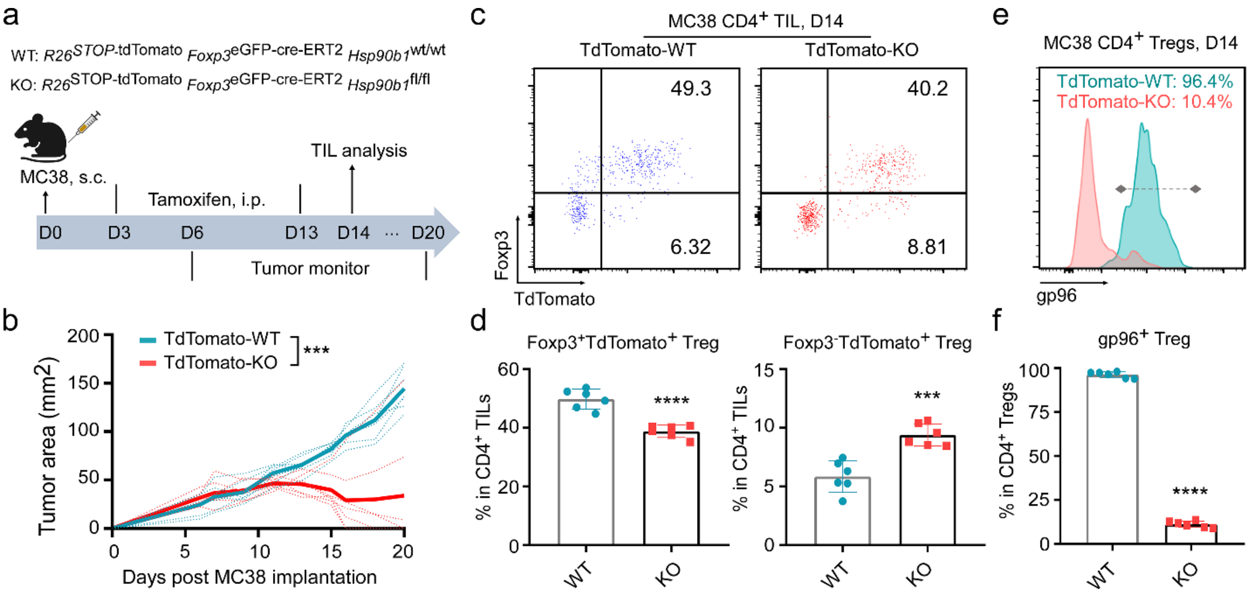

Figure S11. The role of Intratumoral gp96-null Tregs in suppressing MC38 tumor growth in TdTomato-KO mice. a, Experimental schema outlines the process of MC38 implantation ($2x10^6$, s.c.) on day 0 (D0) in TdTomato-WT ($R26^{STOP\text{-}tdTomato}Foxp3^{eGFP\text{-}Cre\text{-}ERT2}Hsp90b1^{wt/wt}$) and TdTomato-KO ($R26^{STOP\text{-}tdTomato}Foxp3^{eGFP\text{-}Cre\text{-}ERT2}Hsp90b1^{fl/fl}$) mice. Tamoxifen treatment was subsequently administrated from D3 to D13 post MC38 implantation. Tumor growth was monitored daily or every two days after MC38 inoculation. On D14, Tumors were collected, and TIL analysis was performed. **b**, Tumor growth curves of MC38 in TdTomato-WT and TdTomato-KO mice (n=7/group). **c-d**, Representative flow cytometry plots (**c**) and summary graphs (**d**) show the percentages (with numbers indicated) of $Foxp3^+tdTomato^+$ and $Foxp3^-tdTomato^+$ Tregs within gated $CD4^+$ TILs on day 14 of MC38 tumors in TdTomato-WT and TdTomato-KO mice (n=6/group). **e-f**, Representative histogram (**e**) and summary graph (**f**) depict the expression (with numbers indicated) of gp96 in gated $CD4^+$ Tregs within day 14 MC38 tumors in TdTomato-WT and TdTomato-KO mice (n=6/group). Results are representative of more than three independent experiments. Data were shown as means ± SEM. ***p<0.001, ****p<0.0001 (KO versus WT). Tumor growth curves were analyzed by repeated measurements two-way ANOVA (S11b). Two-tailed Student's t test was used for comparisons of different experimental groups (S11d, f).

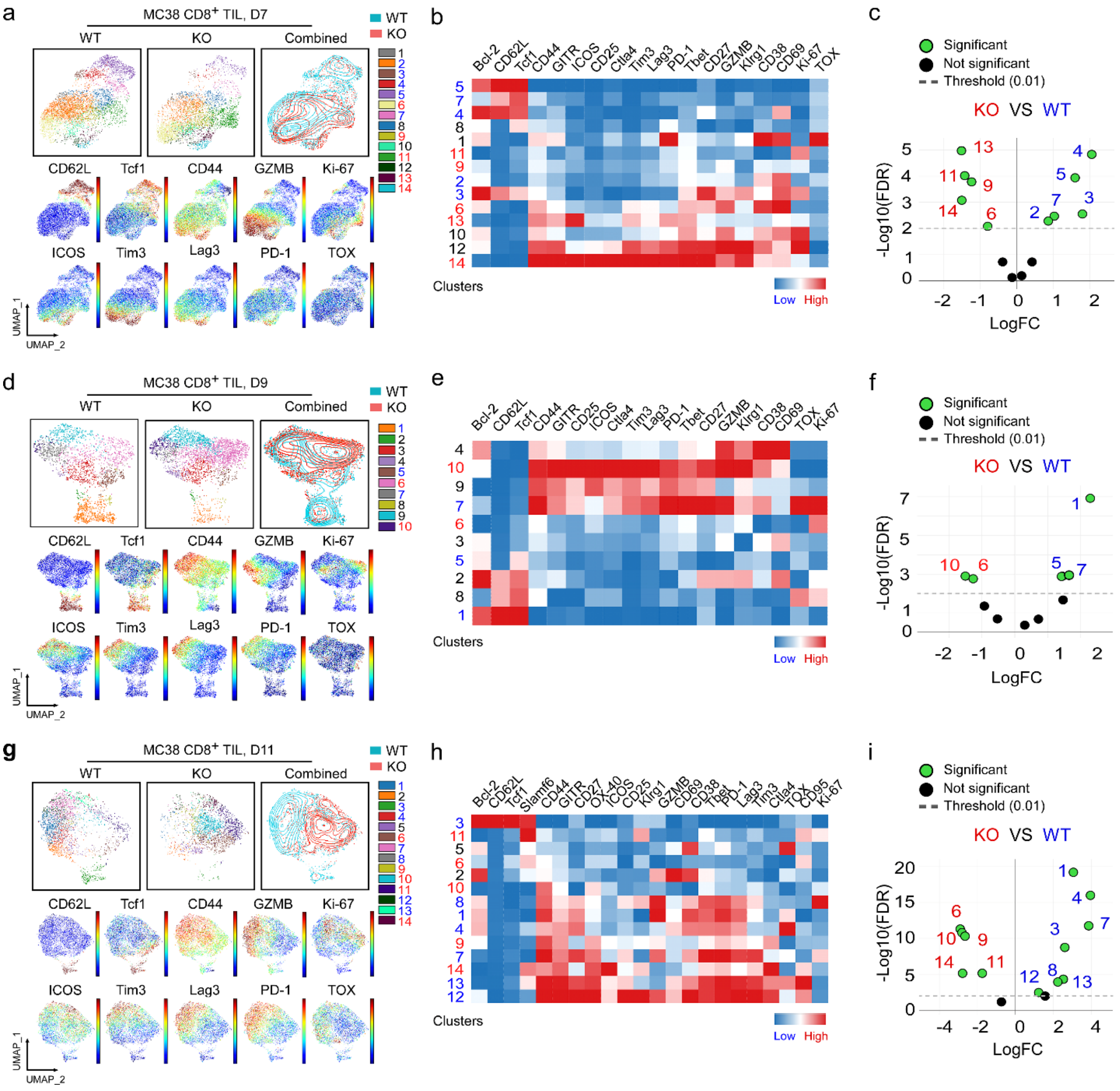

Figure S12. Phenotypical characterization of CD8⁺ TILs in absence of Tregs during MC38 tumors progression. a-c, Spectral flow cytometry analysis of CD8⁺ TILs collected from day 7 (D7) MC38 tumors grown in WT and KO mice (n=7-8/group) receiving tamoxifen treatment (day -10 to 0). Clusters 2, 3, 4, 5, and 7 (subsets of CD44^{low}CD62L^{hi}Tcf1⁺CD8⁺ TILs) were found significantly decreased in the KO group, shown in blue; Clusters 9, 11 (subsets of CD44⁺CD62L^{low}Ki-67⁺CD8⁺ TILs) and clusters 6, 13, and 14 (subsets of CD44^{hi}CD62L^{low}Tcf1⁻ICOS⁺GZMB⁺CD8⁺ effector cells) were found significantly increased in the KO group, shown in red. Expression distribution of specific markers is visualized by UMAP (**at the bottom of a**). **b**, Heatmap visualization of each marker expression by cluster. Cluster numbers were shown on

the left side. **c**, EdgeR analysis indicates clusters significantly enriched in the KO (**left**) versus WT (**right**) group. **d-f**, Spectral flow cytometry analysis of $CD8^+$ TILs collected from day 9 (D9) MC38 tumors grown in WT and KO mice (n=6-7/group) receiving tamoxifen treatment (day -10 to 0). Clusters 1 and 5 (subsets of $CD44^{low}CD62L^{hi}Tcf1^+CD8^+$ TILs) and cluster 7 ($CD44^{hi}CD62L^{low}Tcf1^-TOX^+CD8^+$ TILs) were found significantly decreased in the KO group, shown in blue; Cluster 6 (a subset of $CD44^+CD62L^{low}Ki\text{-}67^+CD8^+$ TILs) and cluster 10 (a subset of $CD44^{hi}CD62L^{low}Tcf1^-ICOS^+GZMB^+CD8^+$ effector cells) were found significantly increased in the KO group, shown in red. Expression distribution of specific markers is visualized by UMAP (**at the bottom of d)**. **e**, Heatmap visualization of each marker expression by cluster. Cluster numbers were shown on the left side. **f**, EdgeR analysis indicates clusters significantly enriched in the KO (**left**) versus WT (**right**) group. **g-i**, Spectral flow cytometry analysis of $CD8^+$ TILs collected from day 11 (D11) MC38 tumors grown in WT and KO mice (n=5-6/group) receiving tamoxifen treatment (day -10 to 0). Cluster 3 (a subset of $CD44^{low}CD62L^{hi}Tcf1^+CD8^+$ TILs) and clusters 1, 4, 7, 8, 12, and 13 (subsets of $CD44^{hi}CD62L^{low}Tcf1^-TOX^+PD\text{-}1^+Tim3^+Lag3^+CD8^+$ TILs) were found significantly decreased in the KO group, shown in blue; Cluster 6, 9, 10, 11, and 14 (subsets of $CD44^{hi}CD62L^{low}Tcf1^-TOX^-PD\text{-}1^-Tim3^-Lag3^-CD8^+$ effector cells) were found significantly increased in the KO group, shown in red. Expression distribution of specific markers is visualized by UMAP (**at the bottom of g)**. **h**, Heatmap visualization of each marker expression by cluster. Cluster numbers were shown on the left side. **i**, EdgeR analysis indicates clusters significantly enriched in the KO (**left**) versus WT (**right**) group. Results are representative of three independent experiments.

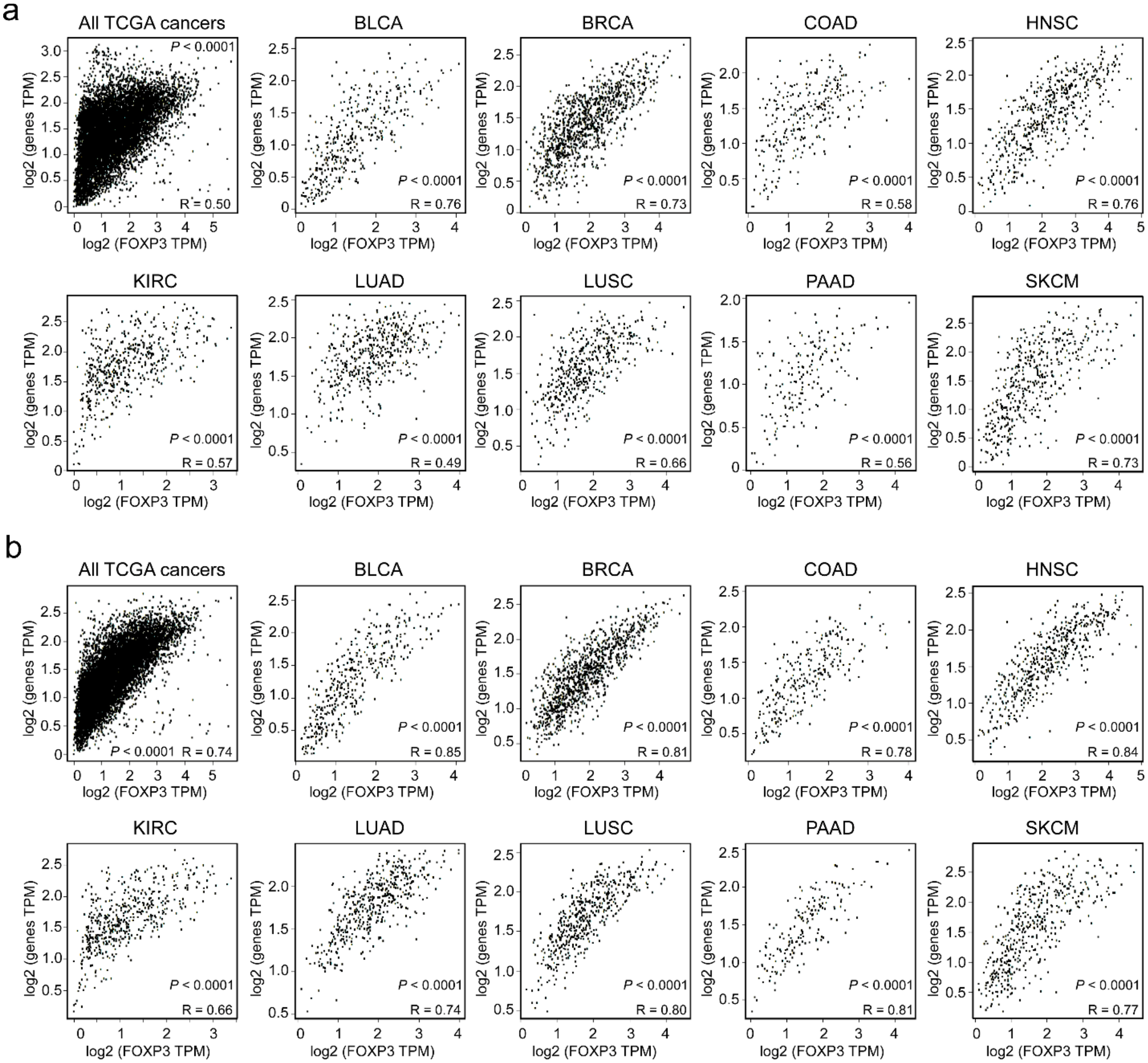

Figure S13. Multi-human cancer analysis of correlation between *FOXP3* and CD8^{+} T cell exhaustion gene signatures. a, Plots showing the correlation between FOXP3, TOX and CD8A mRNA levels among indicated treatment-naïve cancers per TCGA using GEPIA webserver. **b**, Plots illustrating the correlation between mRNA levels of FOXP3 and multiple exhaustion signature genes, including HAVCR2 (TIM3), PDCD1 (PD1), TIGIT, LAG3, CTLA4, CXCL13, TOX, and CD8A across indicated TCGA treatment-naïve cancers using GEPIA webserver. Gene mRNA levels were log2(TPM) transformed. TPM, transcripts per million.

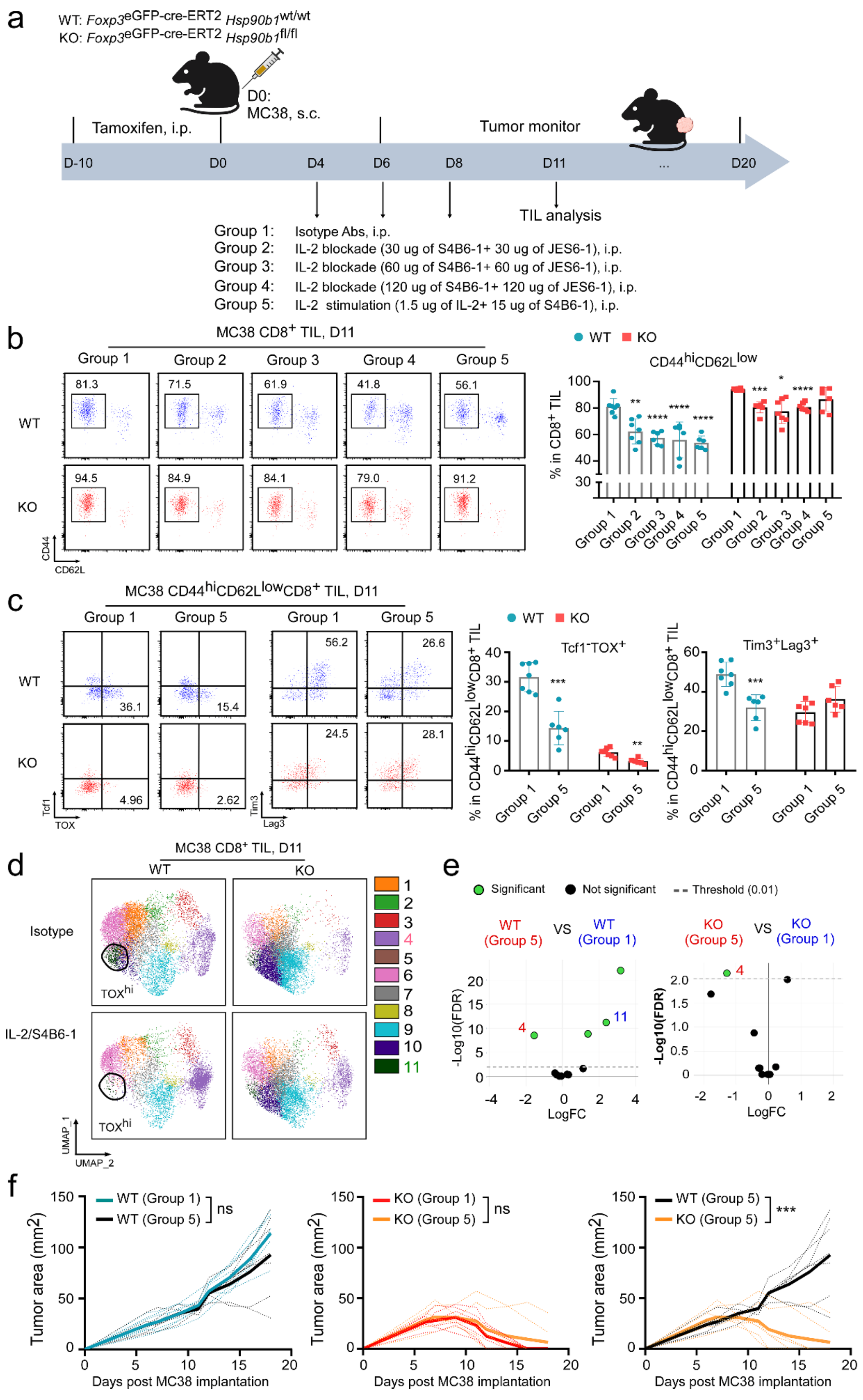
a
WT: Foxp3eGFP-cre-ERT2 Hsp90b1wt/wt
KO: Foxp3eGFP-cre-ERT2 Hsp90b1fl/fl
D0:
MC38, s.c.
Tamoxifen, i.p.
Tumor monitor
D-10
D0
D4
D6
D8
D11
...
D20
TIL analysis
Group 1: Isotype Abs, i.p.
Group 2: IL-2 blockade (30 ug of S4B6-1+ 30 ug of JES6-1), i.p.
Group 3: IL-2 blockade (60 ug of S4B6-1+ 60 ug of JES6-1), i.p.
Group 4: IL-2 blockade (120 ug of S4B6-1+ 120 ug of JES6-1), i.p.
Group 5: IL-2 stimulation (1.5 ug of IL-2+ 15 ug of S4B6-1), i.p.
b
MC38 CD8+ TIL, D11
Group 1
Group 2
Group 3
Group 4
Group 5
WT
KO
81.3
71.5
61.9
41.8
56.1
94.5
84.9
84.1
79.0
91.2
CD44
CD62L
WT
KO
CD44hiCD62Llow
% in CD8+ TIL
c
MC38 CD44hiCD62LlowCD8+ TIL, D11
Group 1
Group 5
36.1
15.4
4.96
2.62
56.2
26.6
24.5
28.1
Tcf1
TOX
Tim3
Lag3
Tcf1-TOX+
Tim3+Lag3+
% in CD44hiCD62LlowCD8+ TIL
d
MC38 CD8+ TIL, D11
WT
KO
Isotype
IL-2/S4B6-1
TOXhi
UMAP_1
UMAP_2
e
Significant
Not significant
Threshold (0.01)
WT (Group 5) VS WT (Group 1)
KO (Group 5) VS KO (Group 1)
-Log10(FDR)
LogFC
f
WT (Group 1)
WT (Group 5)
ns
KO (Group 1)
KO (Group 5)
***
Tumor area (mm2)
Days post MC38 implantation

Figure S14. In presence of Tregs, exogenous IL-2 stimulation prevents TOX-mediated CD8$^+$ TILs exhaustion in MC38 tumors. a, Experimental schema depicts the administration of isotype Abs (group 1), IL-2 blockade Abs (group 2, 3 and 4), or IL-2 stimulation Abs (IL-2/S4B6-1 complexes, group 5) treatment in WT and KO MC38-bearing mice (2x10^6 MC38 cells /mouse, s.c.; n=6-8/group) receiving tamoxifen treatment (day -10 to 0). **b**, Representative flow cytometry plots (**left**) and summary graphs (**right**) show the frequencies (with numbers indicated) of CD44^{hi}CD62L^{low} subset within CD8$^+$ TILs on day 11 (D11) from MC38-bearing WT and KO mice among 5 groups. **c**, Representative flow cytometry plots (**left**) and summary graphs (**right**) illustrate the frequencies (with numbers indicated) of Tcf1$^-$TOX$^+$ and Tim3$^+$Lag3$^+$ in CD44^{hi}CD62L^{low}CD8$^+$ TILs on day 11 from MC38-bearing WT and KO mice between group 1 and group 5. **d**, Spectral flow cytometry analysis of CD8$^+$ TILs isolated from day 11 MC38 tumors grown in WT and KO mice treated with isotype Abs (group 1) or IL-2 stimulation Abs (group 5). Cluster 11 (CD44^{hi}CD62L^{low}Tcf1$^-$TOX$^+$PD-1$^+$Lag-3$^+$Tim-3$^+$CD8$^+$ subset) was cycled and highlighted in green on the right side; Cluster 4 (CD44^{low}CD62L$^+$Tcf1$^+$CD69$^-$CD8$^+$ subset) was found increased by IL-2 stimulation treatment and highlighted in pink on the right side; Expression distribution of indicated markers is shown in **Figure 9d**. **e,** EdgeR analysis indicates the significantly enriched clusters among groups (WT in group 5 versus WT in group 1, KO in group 5 versus KO in group 1). **f**, Tumor growth curves of MC38 (2x10^6) in WT and KO mice receiving isotype Abs (group 1) or IL-2 stimulation Abs (group 5). Tumor sizes were measured as previously described**;** Significance was compared between WT in group 1 versus WT in group 5, KO in group 1 versus KO in group 5, and WT in group 5 versus KO in group 5, respectively. Results are representative of three independent experiments. Data were shown as means ± SEM. ns: no significance, *p<0.05, **p<0.01, ***p<0.001, ****p<0.0001 (IL-2 blockade or IL-2 stimulation versus isotype control). Analyses with multiple comparisons were performed with One-Way ANOVAs with Dunnett's T3 multiple comparisons correction (S14b and S14c). Tumor growth curves were analyzed by repeated measurements two-way ANOVA (S14f).

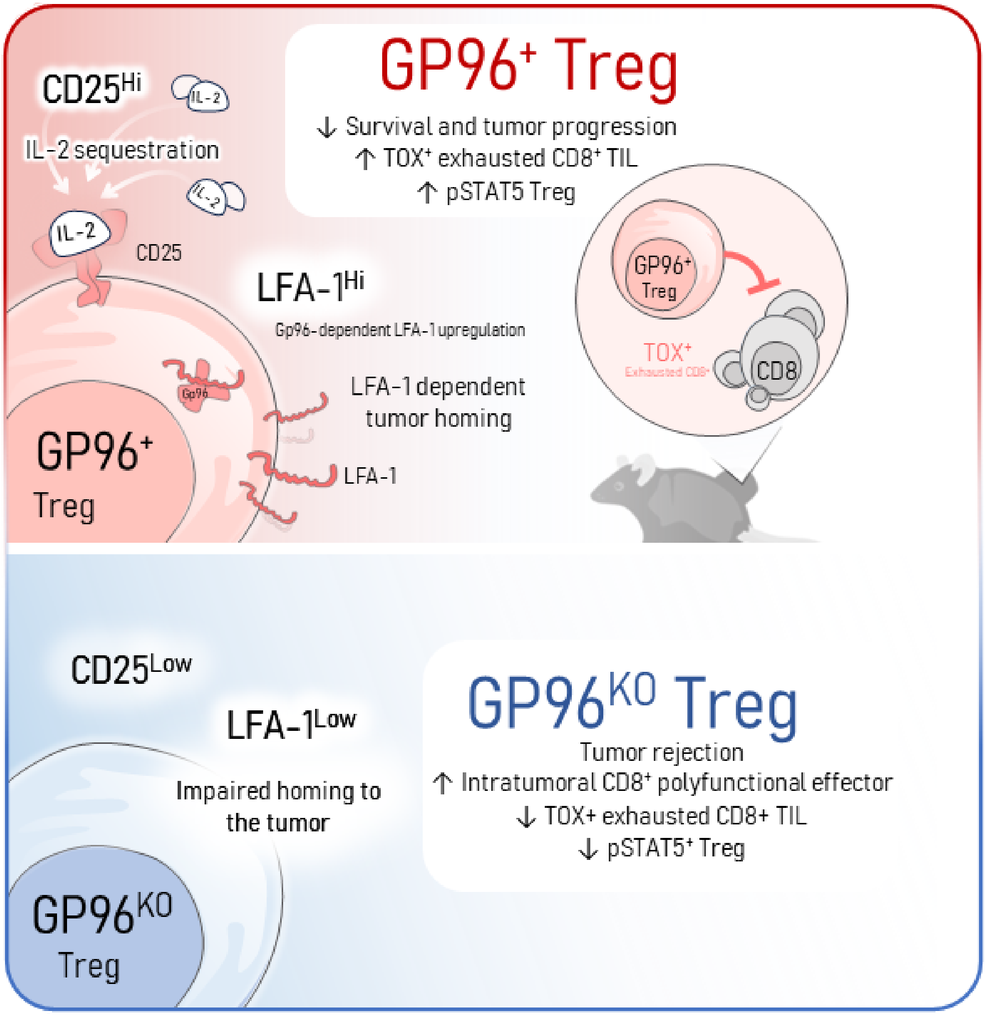

Figure S15. Graphical abstract of findings. Treg migration into the tumor is dependent on the integrin LFA-1 and its chaperone gp96. Deleting gp96/LFA-1 blocks Treg accumulation in the TME and releases intratumoral CD8^{+} T cells from the exhaustion program, resulting in potent anti-tumor immunity.

Extended Tables

Extended Data Table 1. Tumor growth of MB49, MC38 and B16-F10 primary challenge and rechallenge in WT and KO mice.

Extended Data Table 2. Tumor growth of MC38 in in TdTomato-WT and TdTomato-KO mice.

Extended Data Table 3. RNA sequencing analysis of WT and gp96 KO splenic Tregs.

Extended Data Table 4. Tumor growth of MC38 in TdTomato-WT and TdTomato-KO mice subjected to a delayed tamoxifen treatment from day 3 to day 13 following tumor implantation.

Extended Data Table 5. Tumor growth of MC38 in WT and KO mice receiving CD8 depletion and in *Tcrbd*$^{-/-}$ mice adoptively transferred with Tregs and/or CD8^{+} T cells.

Extended Data Table 6. Tumor growth of MC38 in Foxp3^{-DTR} mice receiving PBS or DT treatment.

Extended Data Table 7. Tumor growth of MC38 in WT and KO mice receiving IL-2 antibodies blockade or IL-2/S4B6-1 complexes stimulation.

Extended Data Table 8. Integrin-targeted gRNAs sequences for CRISPR/Cas9 electroporation.

Extended References

1. Levine AG, Mendoza A, Hemmers S, Moltedo B, Niec RE, Schizas M, et al. Stability and function of regulatory T cells expressing the transcription factor T-bet. *Nature.* 2017;546(7658):421-5.
2. Chinen T, Kannan AK, Levine AG, Fan X, Klein U, Zheng Y, et al. An essential role for the IL-2 receptor in Treg cell function. *Nat Immunol.* 2016;17(11):1322-33.
3. McKinstry KK, Strutt TM, Bautista B, Zhang W, Kuang Y, Cooper AM, et al. Effector CD4 T-cell transition to memory requires late cognate interactions that induce autocrine IL-2. *Nat Commun.* 2014;5:5377.
4. Liu Y, Zhou N, Zhou L, Wang J, Zhou Y, Zhang T, et al. IL-2 regulates tumor-reactive CD8(+) T cell exhaustion by activating the aryl hydrocarbon receptor. *Nat Immunol.* 2021;22(3):358-69.
5. Boyman O, Kovar M, Rubinstein MP, Surh CD, and Sprent J. Selective stimulation of T cell subsets with antibody-cytokine immune complexes. *Science.* 2006;311(5769):1924-7.
6. Letourneau S, van Leeuwen EM, Krieg C, Martin C, Pantaleo G, Sprent J, et al. IL-2/anti-IL-2 antibody complexes show strong biological activity by avoiding interaction with IL-2 receptor alpha subunit CD25. *Proc Natl Acad Sci U S A.* 2010;107(5):2171-6.
7. Kamimura D, Sawa Y, Sato M, Agung E, Hirano T, and Murakami M. IL-2 in vivo activities and antitumor efficacy enhanced by an anti-IL-2 mAb. *J Immunol.* 2006;177(1):306-14.
